# Habitat zonation on coral reefs: Structural complexity, nutritional resources and herbivorous fish distributions

**DOI:** 10.1371/journal.pone.0233498

**Published:** 2020-06-04

**Authors:** Arun Oakley-Cogan, Sterling B. Tebbett, David R. Bellwood

**Affiliations:** 1 College of Science and Engineering, James Cook University, Townsville, Queensland, Australia; 2 ARC Centre of Excellence for Coral Reef Studies, James Cook University, Townsville, Queensland, Australia; Department of Agriculture, Water and the Environment, AUSTRALIA

## Abstract

Distinct zonation of community assemblages among habitats is a ubiquitous feature of coral reefs. The distribution of roving herbivorous fishes (parrotfishes, surgeonfishes and rabbitfishes) is a particularly clear example, with the abundance of these fishes generally peaking in shallow-water, high-energy habitats, regardless of the biogeographic realm. Yet, our understanding of the factors which structure this habitat partitioning, especially with regards to different facets of structural complexity and nutritional resource availability, is limited. To address this issue, we used three-dimensional photogrammetry and structure-from-motion technologies to describe five components of structural complexity (rugosity, coral cover, verticality, refuge density and field-of-view) and nutritional resource availability (grazing surface area) among habitats and considered how these factors are related to herbivorous fish distributions. All complexity metrics (including coral cover) were highest on the slope and crest. Nutritional resource availability differed from this general pattern and peaked on the outer-flat. Unexpectedly, when compared to the distribution of herbivorous fishes, none of the complexity metrics had a marked influence in the models. However, grazing surface area was a strong predictor of both the abundance and biomass of herbivorous fishes. The strong relationship between grazing surface area and herbivorous fish distributions indicates that nutritional resource availability may be one of the primary factors driving the distribution of roving herbivorous fishes. The lack of a relationship between complexity and herbivorous fishes, and a strong affinity of herbivorous fishes for low-complexity, algal turf-dominated outer-flat habitats, offers some cautious optimism that herbivory may be sustained on future, low-complexity, algal turf-dominated reef configurations.

## Introduction

The structure of coral reefs is remarkably heterogeneous [1]. Nevertheless, one of the most ubiquitous features of coral reef structure is their partitioning into discrete habitat zones, such as the reef slope, crest and flat [2–4]. Understanding how distribution patterns of coral reef organisms differ among these habitats has been the focus of a burgeoning body of literature [2–6]. Indeed, it is now recognised that the composition of coral and fish assemblages can be more distinct among habitats separated by 10s of meters, compared to assemblages in the same habitat on reefs separated by 1000s of kilometers [7]. The among-habitat distribution of herbivorous fishes (especially parrotfishes, surgeonfishes and rabbitfishes) is particularly well-studied, with remarkably congruent patterns among biogeographic realms. For example, the abundance and biomass of herbivorous fishes is often highest in shallow-water, high-energy habitats such as the crest and flat. This has been reported from the Caribbean [8], Great Barrier Reef (GBR) [4], and Red Sea [9]. However, despite significant advances in our understanding of these patterns, and their potential drivers (e.g. [9–11]), many aspects remain relatively unexplored, including how the structure of these habitats themselves may relate to herbivorous fish distributions.

Structural complexity provides a key interface between an organism and its environment. On coral reefs it can encapsulate topographic complexity [12], predation risk [13] and coral cover [14]. As such, structural complexity and its relationship to reef fishes has received considerable attention in the coral reef literature (e.g. [14–17]). However, few studies have quantified the structure of coral reefs at a scale that fishes use, in the context of its potential influence on fish distributions (but see [13,18,19,20]). In particular, there is a distinct need to disentangle the effects of the different facets of complexity, such as rugosity, refuge density, and their respective influences on a fish’s field-of-view. These different facets have the potential to mediate a range of key drivers of community structure such as predator-prey and/or competitive interactions [21,22], or the influence of physical stressors such as hydrodynamics or UV exposure [23,24].

In addition to the structure of habitats, it has also been posited that the availability of nutritional resources can be a major driver underpinning the distribution of nominally herbivorous reef fishes [10,25–27]. However, habitat structure and nutritional resource availability are not necessarily mutually exclusive and can be intertwined, e.g. more complexity can mean more surface area for fishes to graze [28], along with higher-quality nutritional resources [29]. It is currently unclear how the different facets of complexity differ among distinct reefal zones, how they are inter-related, and what this might mean for the distribution of herbivorous fishes. Understanding the nature and interconnectivity of these structural facets remains a priority in the face of stressors which are expected to reconfigure coral reef systems and push them towards lower complexity systems [12,30]. However, one of the limiting factors in understanding how herbivorous fishes interact with their environment and in teasing apart the relationship between complexity and resource availability, is our ability to accurately measure complexity on coral reefs.

Accurately quantifying three-dimensional (3D) features in marine environments has proven to be a bottleneck for coral reef ecologists for decades. Issues can arise due to: a) differences in methodologies, metrics and scales used to measure complexity among systems (reviewed by [29]); and b) practical and logistical issues associated with identifying, then quantifying, the functional components of complexity on reefs [24]. However, advances in computer vision and camera technologies have allowed underwater researchers to utilise close-range photogrammetry techniques. 3D photogrammetry and structure-from-motion (SFM) technologies now allow researchers to accurately reconstruct computer models of entire sections of reef, covering large spatial extents, in high-resolution (e.g. [30–32]). This 3D technology also facilitates the calculation of a suite of metrics that characterise complexity on coral reefs. However, the application of this technology is still in its infancy and to-date, most studies utilising 3D-methods have been methodological (e.g. [30–32]) rather than being used as a tool to address ecological hypothesis (but see [28,29,33,34]).

If we wish to manage new reef configurations and maintain key ecosystem services, it is vital to understand the nature and role of structure and its effects on key groups of reef organisms. The aims of this study, therefore, are to explore the relationship between habitat structure and roving herbivorous fish (parrotfish, surgeonfish and rabbitfish) distributions across coral reef habitat zones. Based on previous studies, we hypothesised that complexity would be a major driver of fish distributions. However, we can now use novel methods and metrics to elucidate which particular components of complexity exert the strongest influence on fish distributions. To do this, and to measure multiple structural facets simultaneously, we take advantage of recent advances in 3D photogrammetry and SFM techniques to provide new insights into the structure and complexity of coral reef zones and highlight the potential influence of this on herbivorous fish distributions.

## Materials and methods

### Ethics statement

The current study was conducted in accordance with the animal ethics guidelines of James Cook University, Townsville, including authorisation to observe the study organisms under the animal ethics approval number A2529, and the permitting requirements of the Great Barrier Reef Marine Park Authority (permit number: G17/38142.1).

### Study site

**Fig 1:**
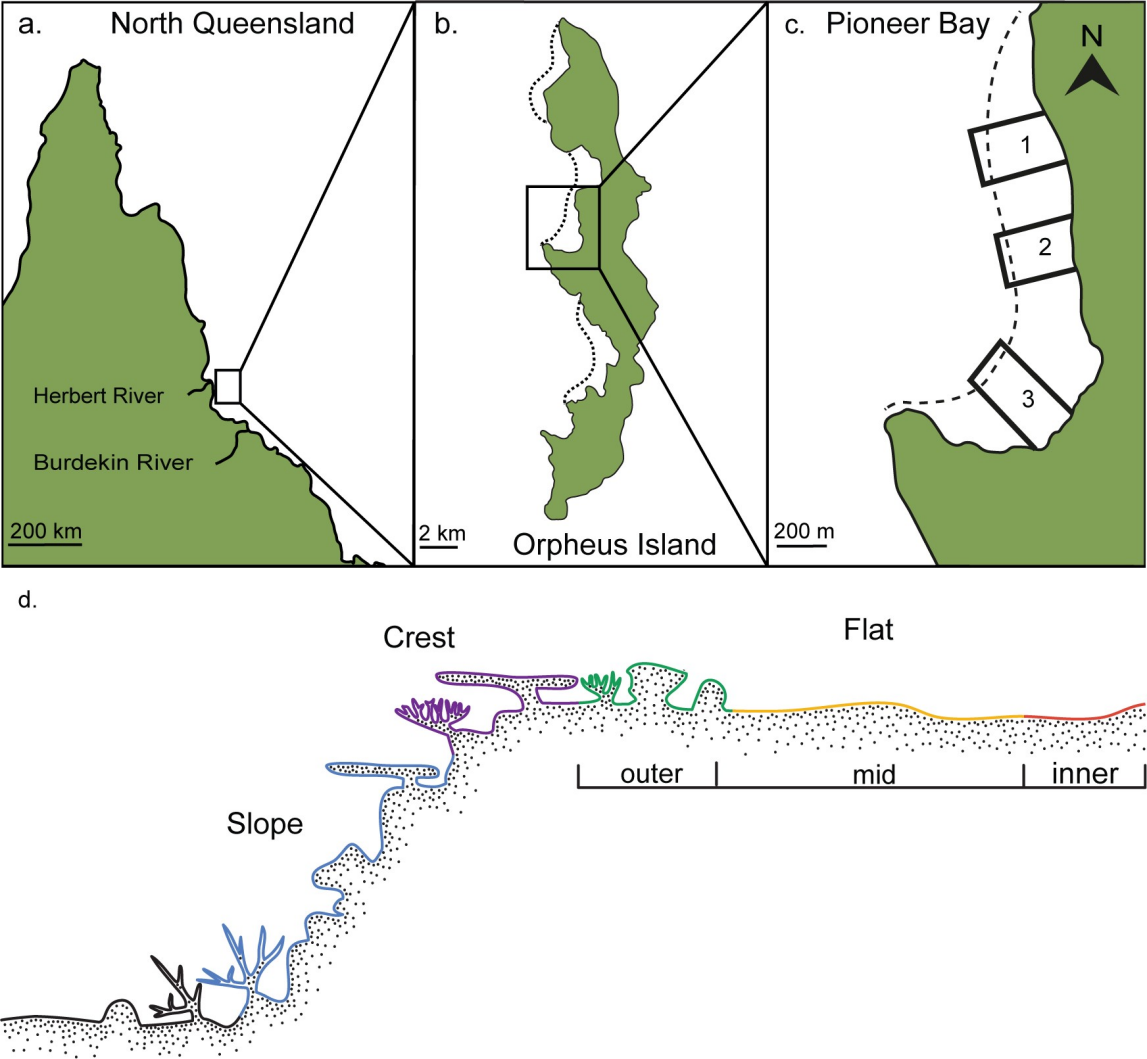
Maps of the survey locations and coral reef gradient on Orpheus Island, Queensland, Australia. a The location of Orpheus Island relative to Queensland, Australia, b Orpheus Island and c the approximate location of the three study sites in Pioneer Bay, Orpheus Island. d A cross-section of a coral reef depth gradient, detailing the major habitat types: reef slope, crest and flat.

This study was conducted during April and May 2018 on reefs in Pioneer Bay, on the leeward side of Orpheus Island (18.618°S, 146.494°E), on the inner-shelf of the GBR (Fig 1). The bay has a clearly delineated reef crest with a reef flat that extends up to 150 m from the shoreline (divided into inner-, mid- and outer-flat habitats [1–3 m water depth]) to the reef crest (1–3 m), which then transitions into the reef slope (3–6 m). These five habitats as described by Fox and Bellwood 2007 [35] form the underlying reef gradient referred to throughout this study. Specifically, the inner-flat was characterized by low coral cover and sandy patches close to the shoreline (1–2 m water depth); the mid-flat was dominated by *Padina* (brown macroalgae) (1–2 m water depth); the outer-flat was consolidated reef matrix and massive *Porites* sp. microatolls, primarily covered in an epilithic algal matrix (EAM) (2–3 m water depth); the reef crest marked the transitional zone between the outer-flat and the slope (2–3 m water depth); the reef slope fell away into deeper water 3–6 m, and was dominated by massive *Porites* colonies (Fig 1D). These five habitats were examined across three study sites within the bay (Fig 1).

### Survey method and 3D reconstructions

To accurately capture the complexity of the benthos along the reef gradient, 3D photogrammetric techniques were utilized. For each reef site and habitat, two replicate 3D reconstructions were taken to describe reef habitat structure across the reef gradient (n = 30; 2 locations per habitat × 5 habitats × 3 sites). To accomplish this, first, video footage was collected with three digital cameras (Nikon Coolpix AW300), each recording at 4K resolutions attached to a custom camera rig (S1A Fig in S1 File). Within each reef flat and crest habitat, a two-diver team collected video footage following the method described by Pizzaro et al. [36], whereby a central fixed drum or ‘survey-station’ was placed at each location, and set 2–2.5 m above the substratum. Attached to the survey-station a 3 m line unwound guiding the surveyor in an expanding circle with constant spacing (15 cm) between revolutions (area covered: ~35 m^2^). Each replicate reef flat and crest habitat was surveyed over two complete passes, using the ‘survey-station’. The first pass required the surveyor to keep all cameras parallel to the substratum in order to have a complete planar view of the underlying area. On the second pass, the cameras were held at an angle of approximately 45°, to increase the amount of vertical data captured. This method ensured that the minimum overlap required in the resulting video footage was obtained, to allow for the reliable generation of 3D reconstructions. However, due to the amount of variation in elevation on the reef slope, the above method could not be applied accurately. To overcome this issue, each slope habitat was marked to delineate a 36 m^2^ area, then a single diver used the more common ‘lawnmower’ method (e.g. [37]), which required the surveyor to follow an approximate grid pattern unaided by external guides. For each slope, three-passes were made by the surveyor. The first two passes required the surveyor to travel both down and up the slope, to establish a planar view. Then, a more ‘freestyle’ pass was used to navigate the vertical regions of each slope to maximise the amount of vertical data captured. To provide a consistent scale in every survey, a system of four dive weights was placed haphazardly around the survey area (prior to surveying). Dive weights were marked with paint at known distances to set a scale for the 3D reconstructions (S1B Fig in S1 File).

**Fig 2:**
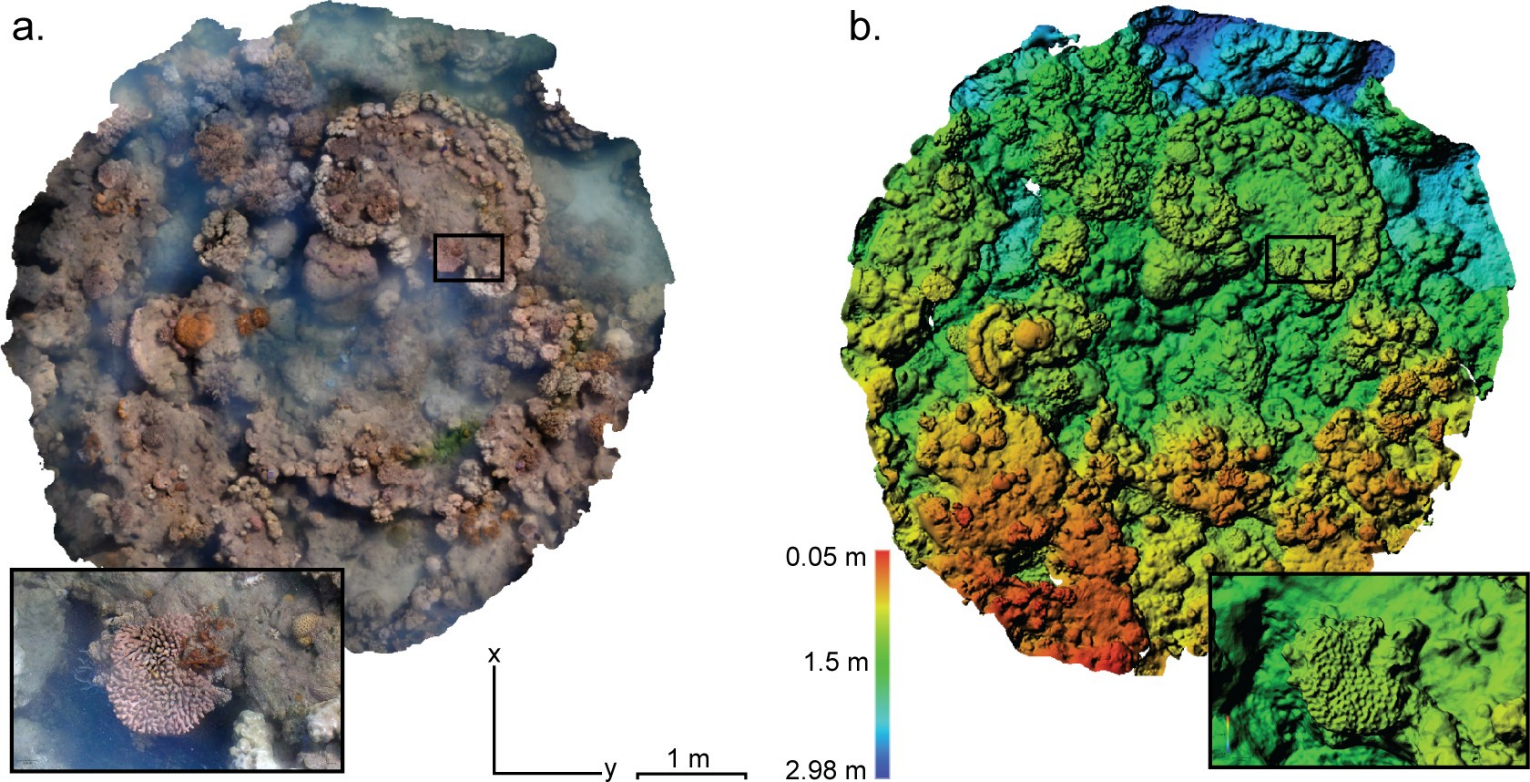
Three-dimensional reconstruction of a reef crest habitat in Pioneer Bay, Orpheus Island. **a** Orthomosaic and **b** digital elevation model of a reef crest section, reconstructed from 2456 images and data cloud of 18,156,733 points. Black bordered insets highlight the separation of height versus appearance in the models and the level of detail produced.

Each video file was converted into image sets by extracting 3 full-resolution frames every second using FFmpeg. Following image extraction, the photogrammetry software Agisoft® PhotoScan Professional v1.4.1 was used to generate 3D reconstructions (3D composite mesh and orthomosaics) of each habitat zone (Fig 2). An upper limit of 40,000 key features was set per image, and a ‘tie-point’ limit of 10,000 was used to constrain the processing time (following the recommendations of Agisoft 2018). All summary statistics for the 3D models were gathered using Agisoft® PhotoScan Professional v1.4.1. Please refer to S13 Table in S2 File for all Photoscan parameters.

### Complexity metrics

To collect complexity metrics, five randomly generated 5 m transects were overlayed on each 3D reconstruction. Then, for each transect, the topographic profile cross-section was calculated (S2 Fig in S1 File). Using these cross-sections and the orthomosaics of reef structure, the following complexity metrics were obtained: a) rugosity index, b) average rate of change in elevation (verticality), c) hard coral cover, d) available refuges, e) feeding field-of-view and f) grazing surface area. Each of these metrics is obtained from five independent (1-D) transects; in effect we were applying traditional liner 1-D measures of complexity within a 3D digital reconstruction. Each metric is described in detail below, all measurements were taken within Agisoft® PhotoScan Professional.

### Rugosity index

The rugosity index (RI) is a standard measure of terrain roughness, comparing the heterogeneous surface length to its planar distance. Typically, in coral reef studies, this has been accomplished through the chain-and-tape method, whereby a chain is draped to lie over the contours of a surface (*D_chain_*) and compared to its linear distance (*L_chain_*) (S2 Fig in S1 File). Virtual chain-and-tape measures were taken using the 5 m transects generated for each plot, where both the virtual chain length and distance covered were calculated to give an RI.

$$RI=\frac{L_{chain}}{D_{chain}}$$

### Verticality

As another proxy for complexity, the average rate of change (*A*(*x*)) in verticality was calculated along each cross-section. The average rate of change calculates the amount of change in vertical height in relation to distance along each transect (S3 Fig in S1 File). This metric was calculated at 10 cm intervals along each reef cross-section. 10 cm intervals were chosen as fishes tend to associate with complexity relevant to their body size [38] and 10 cm is approximately the mean body size of visually apparent reef fishes [39]. The metric was calculated by taking the absolute value of the bathymetric height at the beginning (*f*(*a*)) and end (*f*(*b*)) of each 10 cm interval along the cross section and then dividing by the interval distance:

$$A(x)=Abs\left(\frac{f(b)-f(a)}{10cm}\right)$$

### Hard coral cover

The distribution and abundance of reef fishes has often been linked to the abundance of live coral cover [40–42], with some studies finding positive correlations between the amount of live coral cover and reef fish assemblages [43,44]. Furthermore, coral cover is often viewed as a proxy for complexity with numerous studies showing a positive correlation between the two (reviewed by [12]). Using the reconstructed orthomosaics, the proportion of hard coral cover along each transect was estimated by highlighting each section of transect covered in live coral, accounting for areas of partial mortality. The contoured profile of the highlighted section could then be quantified and measured (S4 Fig in S1 File).

### Refuge density

Crevices and overhangs can provide areas of physical refuge from predation [16,24,38,41]. Herein, we defined a physical refuge as being any crevice that could fit the average size of herbivorous fishes in this study (crevice height: min 10 cm, width: 10–20 cm). As the 3D reconstructions were not able to capture the depth of overhangs on topographic cross-sections, an overhang was defined as any vertical surface greater than 10 cm high. The density of crevices and overhangs were counted directly from the cross-sectional transects by using a circle set at a 10 cm diameter, representing a cross-section of a fish (S5 Fig in S1 File).

### Feeding field of view

Typically, viewshed is used in geographic planning and management to estimate the proportion of terrain visible from a given location (e.g. fire towers) [45]. Previously, studies have used this standard concept of viewshed as a proxy for predation risk [28,46]. In this respect it is assumed that exposure to predation relates directly to the degree of openness for that particular section of reef. In this study, a different approach was used to more accurately capture predation risk. This new approach centred on the potential for a herbivorous fish to see a predator whilst feeding by estimating the proportion of available viewing area during feeding activities. A period when fish appear to be most susceptible to predatory events [47].

Firstly, reef cross-sections from each virtual chain-transect, were imported and scaled in the software ImageJ [48]. Then, observer dots representing mean fish eye height while feeding (2.5 cm above the benthos [49]), were placed at 1 m intervals along the length of the transect, starting from 0.5 m. From each of the observer points, a horizontal line was drawn from each observer point inwards towards the middle of the reef profile (to gauge the amount of blocked terrain in both directions), with the exception of the middle point in each transect, where horizontal lines were drawn in both directions (S6 Fig in S1 File). The horizontal line was set at a length of 2.5 m. This length was chosen based on average herbivorous reef fish flight initiation distance [50,51] and water visibility in the given location (S10 Table in S2 File). Finally, the magnitude of the angle from the horizontal line to a secant drawn touching the highest elevation point in the terrain was subtracted from 90 degrees to yield the proportion of blocked field of view.

### Grazing surface area

Most nominally herbivorous fishes on coral reefs feed on one or more components of algal turfs or EAM, a highly productive and nutritious resource [52–55]. For the purposes of this study, grazing surface area was considered to consist of any hard, consolidated surface (e.g. reef matrix, dead coral) that was covered in short (< 2 cm high) algal turfs. Macroalgae was not considered in grazing surface area calculations because only a few herbivorous fishes feed heavily on macroalgae [56–58]. The proportion of the substrate covered in algal turfs (EAM) (i.e. potential grazing area) was calculated using the same procedure as used for estimating hard coral cover (S7 Fig in S1 File).

### Environmental data

To capture key environmental conditions across the reef gradient, in addition to complexity metrics, both average water depth and tidal effects were quantified. For depth, nine replicate measurements (to 0.1 m) were taken for each habitat in each site. A 2 m depth gauge was used in the flat and crest habitats and a 5 m graduated rope was used on the slope. All depth measurements were taken within one hour of high tide, and the ‘Rule of Twelfths’ applied, to approximate sine curves and therefore estimate the height of the tide at any time, given the time of day and height of high/low water [59] (S11 Table in S2 File). To consider the effects of tides on the ability of herbivorous fishes to graze the reef flat we calculated the proportion of grazing time per year that access to the reef flat would be limited by water depth [3]. To do this we used tide data for the Lucinda (offshore) region (18° 53´ S, 146° 33´ E) in the GBR, Australia for the year leading up to this study [60]. We calculated the percentage of time per year that each reef habitat was covered by < 30 cm thereby excluding roving herbivorous fishes from grazing (S12 Table in S2 File).

### Roving herbivorous fish surveys

To quantify the abundance of roving herbivorous fishes (parrotfishes [Labridae; Scarini], surgeonfishes [Acanthuridae] and rabbitfishes [Siganidae]), a series of five-minute timed swims were used. For each swim the first diver recorded fishes >10 cm total length (TL) in a 5 m wide transect and the second diver recorded fishes <10 cm TL in a 1 m wide transect (following [61]). Both divers placed fishes into TL size categories: 5 cm intervals for fishes >10 cm TL and 2.5 cm intervals for fishes <10 cm TL. The length of each transect was calibrated using GPS locations (marked at the start and end of each transect). The average length (± SE) of each transect was 47.25 m ± 2.21 m. Each census was repeated three times on separate days (within 1 hour of morning high tide), to provide a more robust estimate of average fish distributions. Fish biomass was subsequently calculated using Bayesian length-weight regression parameters for each species from Fishbase [62].

### Statistical analysis

#### Modelling reef fish and complexity across habitats

Complexity metrics, fish abundance and biomass were initially compared among habitat zones using Bayesian generalised linear mixed-effects models. In all cases habitat (levels = slope, crest, outer-, mid-, inner-flat) was considered the sole fixed effect. Beta-binomial distributions with a logit link were used to analyse proportional data (i.e. hard coral cover and grazing surface area). As the beta-binomial distribution is bounded by 0 and 1 a small constant was added (0.001) to all data to account for zeros in the data set (Table 1) [63]. Gaussian distributions were used to model continuous metrics, i.e. rugosity, verticality and field-of-view (Table 1). A Gamma distribution (with a log link) was used to model the continuous, strictly positive fish biomass data (Table 1). While for count data (refuge density and fish abundance), negative binomial distributions were used because of the non-normally distributed and overdispersed nature of the data (Table 1) [64]. To account for any lack of spatial dependence, all models included site as a random factor.

**Table 1:** Summary of model equations and distributions used to compare complexity metrics: Rugosity, verticality, hard coral cover, refuge density, field-of-view, grazing surface area and herbivore abundance and biomass among coral reef habitats (i.e. slope, crest, outer-, mid- and inner-flat) along a depth gradient.

Model equation	Model distribution
Rugosity ~ Habitat + (1|SITE)	Gaussian (identity link)
Verticality ~ Habitat + (1|SITE)	Gaussian (identity link)
Hard coral cover ~ Habitat + (1|SITE)	Beta-binomial (logit link)
Refuges ~ Habitat + (1|SITE)	Negative binomial (log link)
Field-of-view ~ Habitat + (1|SITE)	Gaussian (identity link)
Grazing surface area ~ Habitat + (1|SITE)	Beta-binomial (logit link)
Herbivore abundance ~ Habitat + (1|SITE)	Negative binomial (log link)
Herbivore biomass ~ Habitat + (1|SITE)	Gamma (log link)

#### Model assumptions

Model fits and assumptions were assessed using trace, autocorrelation and residual plots, accompanied by assessments of sampling efficiency (rhat) and effective sample size (Neff) scores, all chains were well-mixed, and plots were satisfactory.

#### Model interpretations

Each model was based on 3 chains with 5000 iterations, including a warm-up of 2500 iterations and a thinning interval of 3, with weakly informative priors (S14 Table in S2 File). To examine differences among individual habitat zones, multiple comparisons were used. Inferences were based upon the mean slope of the predictor variable and associated 95% high posterior density intervals (HPDI). If the HDPIs intersected zero, no effect was inferred.

#### Modelling herbivorous reef fish populations

Following the examination of patterns among habitats, the relationship between complexity metrics and herbivorous reef fish abundance and biomass was formally examined. Initially, pair plots and Pearson’s correlation coefficients among the complexity metrics were assessed to examine collinearity among the complexity metrics (S8 Fig in S1 File). For complexity metrics that were distinctly collinear (> ± 0.7 Pearson’s correlation) [64,65] a principle co-ordinate analysis was constructed based on a dataset of normalised metrics. Subsequently, vector loadings on the first principal component were computed and used as a combined complexity covariate in models [66]. Based on the set of continuous covariates (each of the complexity variables and depth), full additive models were initially fitted with herbivorous fish abundance and biomass as dependent variables.

To match herbivorous fish abundance/biomass to the complexity metrics at the same scale, mean values for each habitat within each site (n = 15), were used in the models. Site was fitted as a random factor to account for any lack of spatial independence. As to not overfit Bayesian models, the most parsimonious model was obtained for both herbivorous fish abundance and biomass based on the leave-one-out-cross-validation (LOO) method (S1 Table in S2 File) [67]. Both Bayesian models (based on fish abundance or biomass) were fitted using Gamma distributions and a log link with 3 chains, 5000 iterations, a warm-up of 2500 iterations and a thinning interval of 3, with weakly informative priors (S14 Table in S2 File). The assumptions of final models were assessed as above (S1 Table in S2 File). All statistical analyses were conducted within R [68] using the *glmmTMB* [69], *lme4* [70], *brms* [71], *car* [72], *rstanarm* [73] and *emmeans* [74] packages.

In addition to examining the entire roving herbivorous fish assemblage, multivariate analyses were also used to examine species-specific relationships. Patterns in the fish species assemblage were initially visualised among habitats using non-metric multidimensional scaling (nMDS) ordination plots. Both the abundance (divided into small [<10 cm] and large [>10 cm] individuals) and biomass of the herbivorous fish species assemblage were examined. In both cases the nMDS plots were based on zero-adjusted Bray-Curtis similarity matrices. Following this, correlations between the multidimensional data clouds for fishes, and metrics (as above) were formerly examined using distance-based linear models. Model outputs were subsequently visualised using distance-based redundancy analysis. Multivariate analysis was performed in Primer V7 PERMANOVA+.

## Results

The methods used in this study efficiently and accurately captured the variation in the structure of the habitats examined across large spatial areas. Each pass of a habitat, to capture video footage, took on average 3.76 ± 0.07 minutes (± SE) and covered an area of 41.89 ± 1.03 m^2^ (mean ± SE). This short amount of time spent underwater was all that was necessary to reconstruct models with a large degree of accuracy (although the reconstruction of each model took between 3 to 7 days). Reprojection errors were remarkably low: 1.14 ± 0.08 pixels, with each pixel having a ground resolution of 0.33 ± 0.01 mm. Errors resulting from scaling were also extremely low: 1.43 ± 0.0003 mm (mean ± SE). Essentially, the habitats in Pioneer Bay were reconstructed in fine detail (to less than 1 mm), providing an unprecedented ability to examine various complexity metrics without the time constraints of scuba.

### Complexity metrics among reef habitats

**Fig 3:**
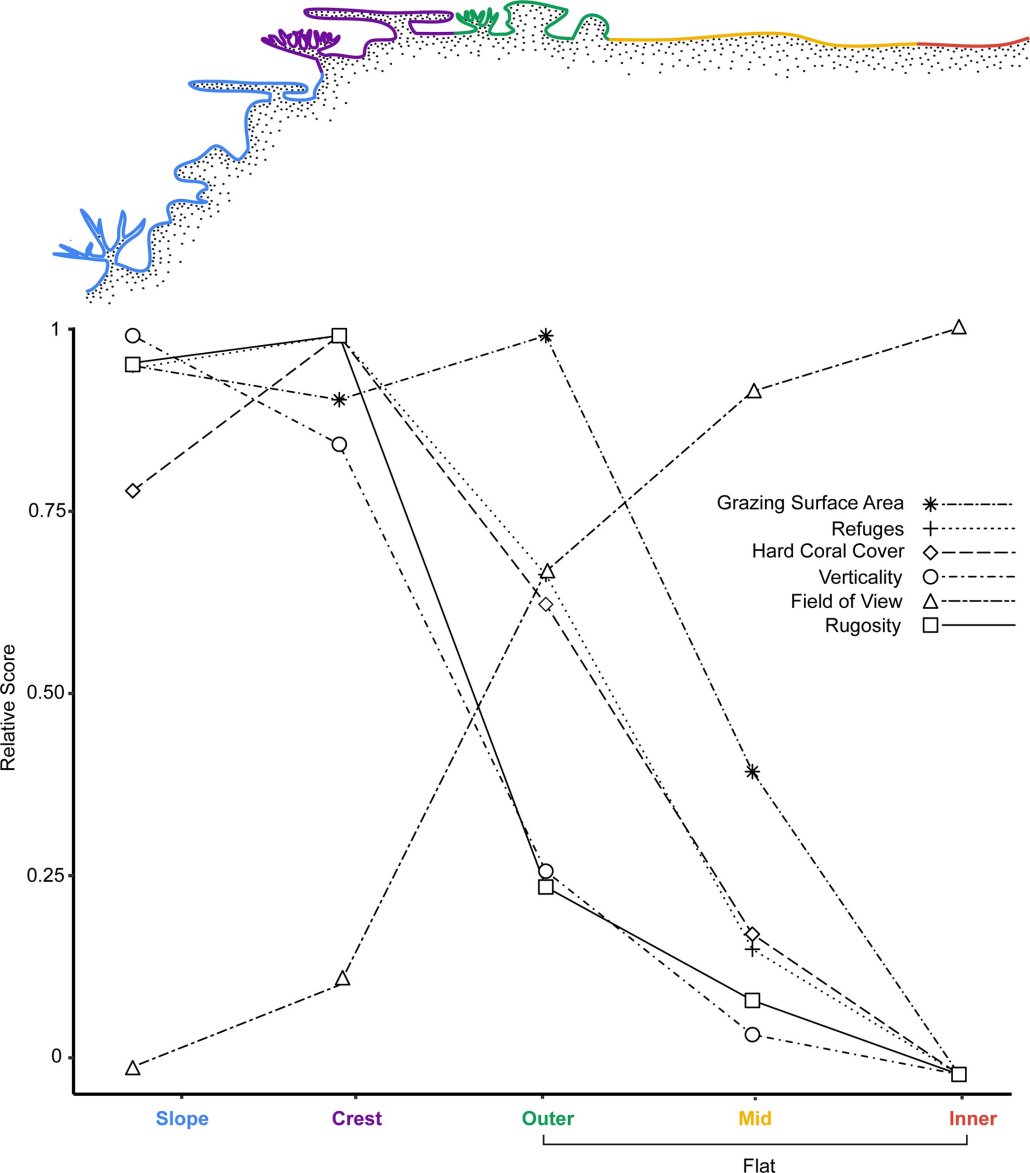
Complexity metrics along a coral reef depth gradient in Pioneer Bay, Orpheus Island.

Complexity metrics were markedly different among habitats. Specifically, in all sites, the reef slope and crest were the most complex habitats, with complexity decreasing across the flat towards the shoreline (Fig 3). For RI, the slope and crest were 38.7% higher, on average, than for the flat habitats (S9 Fig in S1 File). While the slope and crest did not differ substantially from one another in their effect sizes (the crest was 60% more likely to have higher RI values than the slope). With both the crest and slope displaying higher RI values compared to all flat habitats (S2 Table in S2 File; S2 Fig in S1 File). Differences among flat-habitats were minimal, with only a 10.7% decrease in RI between the outer- and inner-flat habitats (S3 Table in S2 File).

All complexity metrics were quantified using 3D habitat reconstructions. Note the congruence in patterns among the first 4 complexity metrics and the divergence of grazing surface area and field-of-view from this pattern (see S9 Fig in S1 File for specific complexity metrics)

At an increased scale, when verticality was considered, differences in complexity produced similar patterns to those for RI (Fig 3; S2 Table in S2 File). Indeed, the only difference with verticality was an increase in values between the slope and crest (S3 Table in S2 File). Patterns for hard coral cover were also remarkably similar to RI and verticality (Fig 3; S2 Table in S2 File). As expected, the crest was the area of highest coral cover (36.1%, -0.008, + 0.007) (mean %, -lower HPDI, + upper HPDI) (S9 Fig in S1 File).

Although refuge density was measured at a different scale, using a very different technique to all other complexity metrics, again there was remarkable congruence in the observed pattern (Fig 3). Essentially, refuge density was highest on the slope and crest, and decreased across the flat towards the shoreline (Fig 3; S4 and S5 Tables in S2 File). The congruence among RI, verticality, hard coral cover, and refuge density all suggest that the crest, and to a lesser extent the slope, offered the highest complexity areas, across the spatial scales examined.

In a direct inverse relationship to the previous four metrics, however, the potential field-of-view for fishes was greatly reduced on both the slope and crest, when compared to all three reef flat habitats (Fig 3; S4 and S5 Tables in S2 File). Indeed, a fish grazing on the reef crest could see 19% less of their surrounding environment, on average, compared to a fish grazing on the inner-flat. Taken together, this suggests that the ability of fishes to see predators on the crest and slope was lower than on the flat, however, there was a far higher chance of finding suitable refuges.

The greatest divergence from these patterns, was in the available grazing surface area (Fig 3; S9 Fig in S1 File). While grazing surface area was still high on the slope (54.5%, - 0.02, + 0.02) and crest (51.5%, -0.02, + 0.02), it was highest on the outer-flat (57.3%, -0.02, + 0.02). While grazing surface area was not substantially different among the slope, crest and outer-flat, it did differ with high probability between these three habitats and the mid- and inner-flat (S9 Fig in S1 File; S5 Table in S2 File). For example, the average cover of grazing surface area was 48.6% higher on the outer-flat than on the inner-flat.

The six different metrics, therefore, revealed three distinct characteristic habitat clusters in terms of predation risk/refuge availability and nutritional resource availability. The slope and crest were typified by high complexity and high nutritional resource availability, but a low feeding field-of-view. The outer-flat was typified by high nutritional resource availability and a high feeding field-of-view with low complexity. While the mid- and inner-flat were typified by low nutritional resource availability and complexity but a high feeding field-of-view (Fig 3).

Finally, environmental data showed expected trends in exposure across the slope and crest as these habitats were accessible to herbivorous fishes 100% and 99.4% of the year, respectively. Remarkably, the outer-, mid- and inner-flat habitats were also widely accessible for 97.3%, 94.4% and 87.2% of the year, respectively.

### Roving herbivorous fish distributions

A total of 13 species of roving herbivorous fishes were recorded during the visual surveys (S6 Table in S2 File). However, their abundance and biomass distributions were dominated by three species (*Scarus rivulatus*, *Siganus doliatus* and *Chlorurus microrhinos*). Parrotfishes contributed the most in terms of abundance (100.9 ± 10.4 ind. 250 m^-2^, mean ± SE) and biomass (9.17 ± 2.2 kg 250 m^-2^) making up more than two thirds of the local herbivorous fish assemblage (abundance: 77.2% and biomass: 72.5%). This was followed by rabbitfishes (abundance: 15.7% and biomass: 20.9%) and finally surgeonfishes (abundance: 7% and biomass: 6.6%).

**Fig 4:**
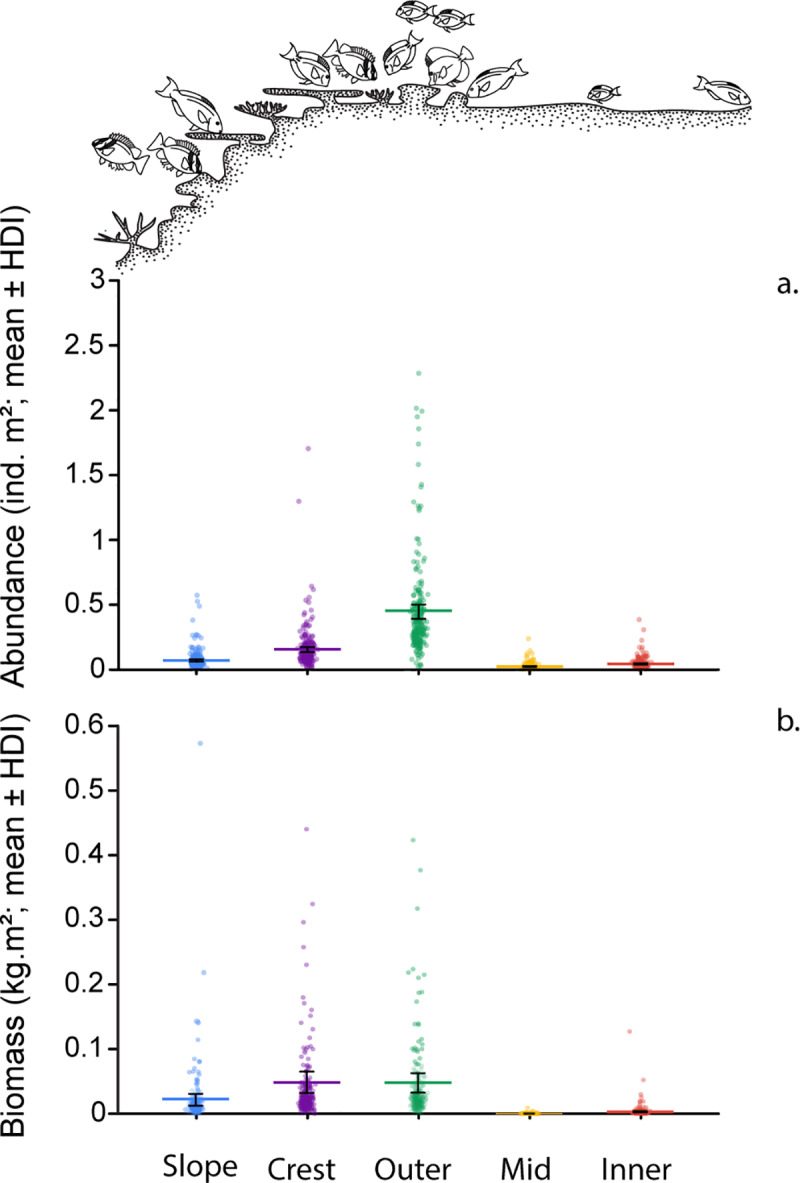
The distribution of roving herbivorous fishes (parrotfishes, surgeonfishes and rabbitfishes). **a** abundance and **b** biomass, across the reef depth gradient in Pioneer Bay, Orpheus Island. Data points represent a random sample of 250 draws from the modelled posterior distribution of Bayesian models. Coloured bars represent back-transformed mean values from posterior distributions and error bars are the lower and upper high posterior density intervals.

The abundance of roving herbivorous fishes varied greatly across the reef gradient and peaked on the outer-flat followed by the crest (S7 Table in S2 File; Fig 4A). Indeed, the mean abundance of herbivorous fishes on the outer-flat (0.34 ind. m^-2^, -0.038, + 3.6) (mean,—lower HPDI, + upper HPDI) was more than 2.8-, 6.5-, 9.6- and 53.4-fold higher than on the crest, slope, inner-flat and mid-flat, respectively (Fig 4A). Fish biomass also showed considerable differences among habitats (S7 Table in S2 File; Fig 4B). Again, biomass was highest on the outer-flat (0.043 kg m^-2^, -0.024, +0.06), although this did not differ from the crest with a high degree of probability (0.035 kg m^-2^, -0.029, + 0.041). However, the biomass of herbivorous fishes on the outer-flat was more than 2.9-, 20.1- and 114.1-fold higher than on the slope, inner-flat and mid-flat, respectively (Fig 4B). Once again, these were all conferred with high degrees of probability (S8 Table in S2 File). Thus, the outer-flat, and to a lesser extent the crest, were the preferred habitats for roving herbivorous fishes in Pioneer Bay.

### Relationship between complexity metrics and herbivorous fishes

**Fig 5:**
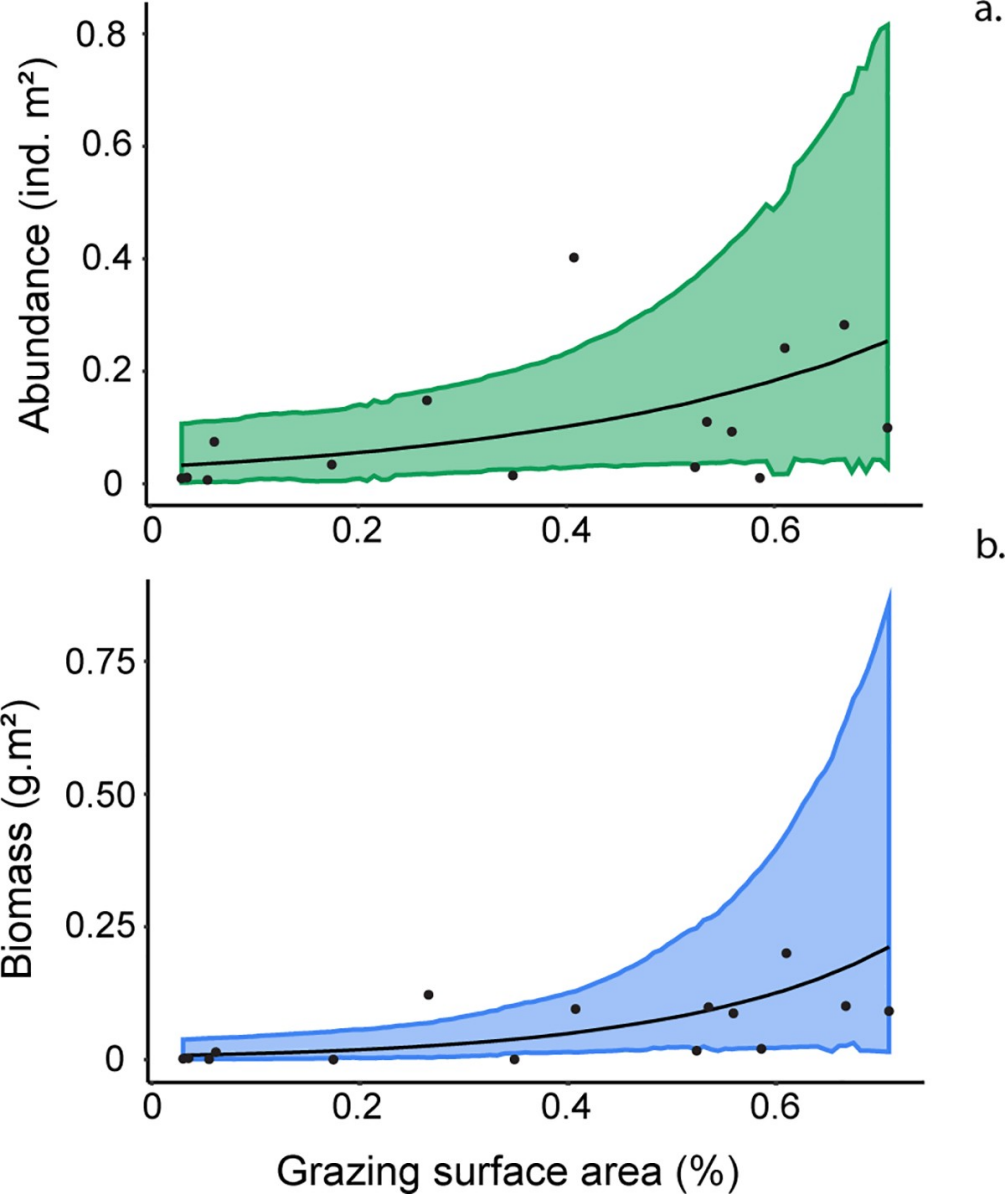
The relationship between roving herbivorous fishes and grazing surface area. The **a** abundance and **b** biomass of roving herbivorous fishes and grazing surface area in Pioneer Bay, Orpheus Island. The predicted line shows the predicted fit from Gamma distributed Bayesian hierarchical models and their upper and lower 95% high posterior density intervals.

Due to the high degree of congruence in the patterns of the first five metrics (RI, verticality, hard coral cover, refuge density and field of view) these metrics were all found to be highly co-linear (Pearson’s correlation > ± 0.7; S1 Fig in S1 File). Likewise, habitat exposure was found to be co-linear with grazing surface area, but as fish surveys were conducted when all habitats were accessible to herbivorous fishes, this metric was dropped from final models. Therefore, principal component 1 (PC1) was selected as the representative complexity metric in our models (Fig 4), along with the non-co-linear variable, grazing surface area (nutritional resource availability) and depth. Based on the LOO selection, the most parsimonious model for abundance contained only grazing surface area as a factor. While the selected model for biomass included grazing surface area, depth and complexity as factors, only grazing surface area had a substantial effect (S1 Table in S2 File). Therefore in both cases, final models suggested that there was a >95% chance that grazing surface area was positively correlated with the distribution of herbivorous fish abundance (S9 Table in S2 File; Fig 5A) and biomass (S9 Table in S2 File; Fig 5B).

**Fig 6:**
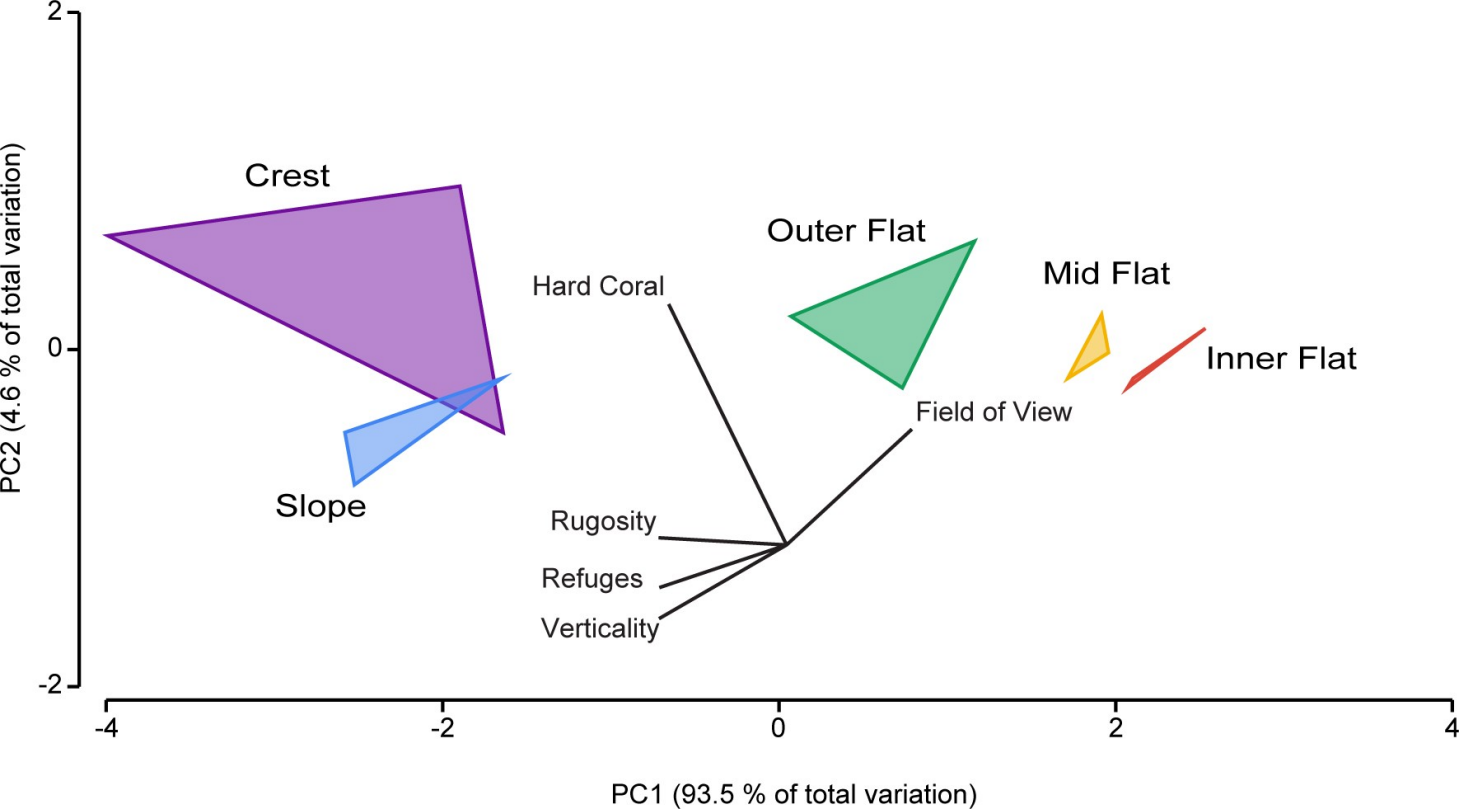
Principle co-ordinate analysis of five complexity metrics across a reef depth gradient in Pioneer bay, Orpheus Island.

In terms of the species-specific distance based linear model analyses, a few species displayed particularly notable patterns. Both the abundance (>10 cm) and biomass of the parrotfish *S*. *rivulatus* were distinctly correlated with the coverage of grazing surface area and both were strongly associated with the outer-flat habitat and to a lesser extent the crest (Fig 6). In addition, the abundance (> 10 cm) and biomass of the rabbitfish *S*. *doliatus* was correlated with grazing surface area and areas of increasing complexity, associated primarily with the crest habitats (Fig 6). By contrast, the abundance (>10 cm) and biomass of *C*. *bleekeri* and *Si*. *lineatus* were strongly correlated with deeper habitats (e.g. slope).

Complexity metrics reveal their correlation amongst each other and their relationship to various habitats. Coloured polygons are to aid visual interpretation and do not denote significant groupings.

**Fig 7:**
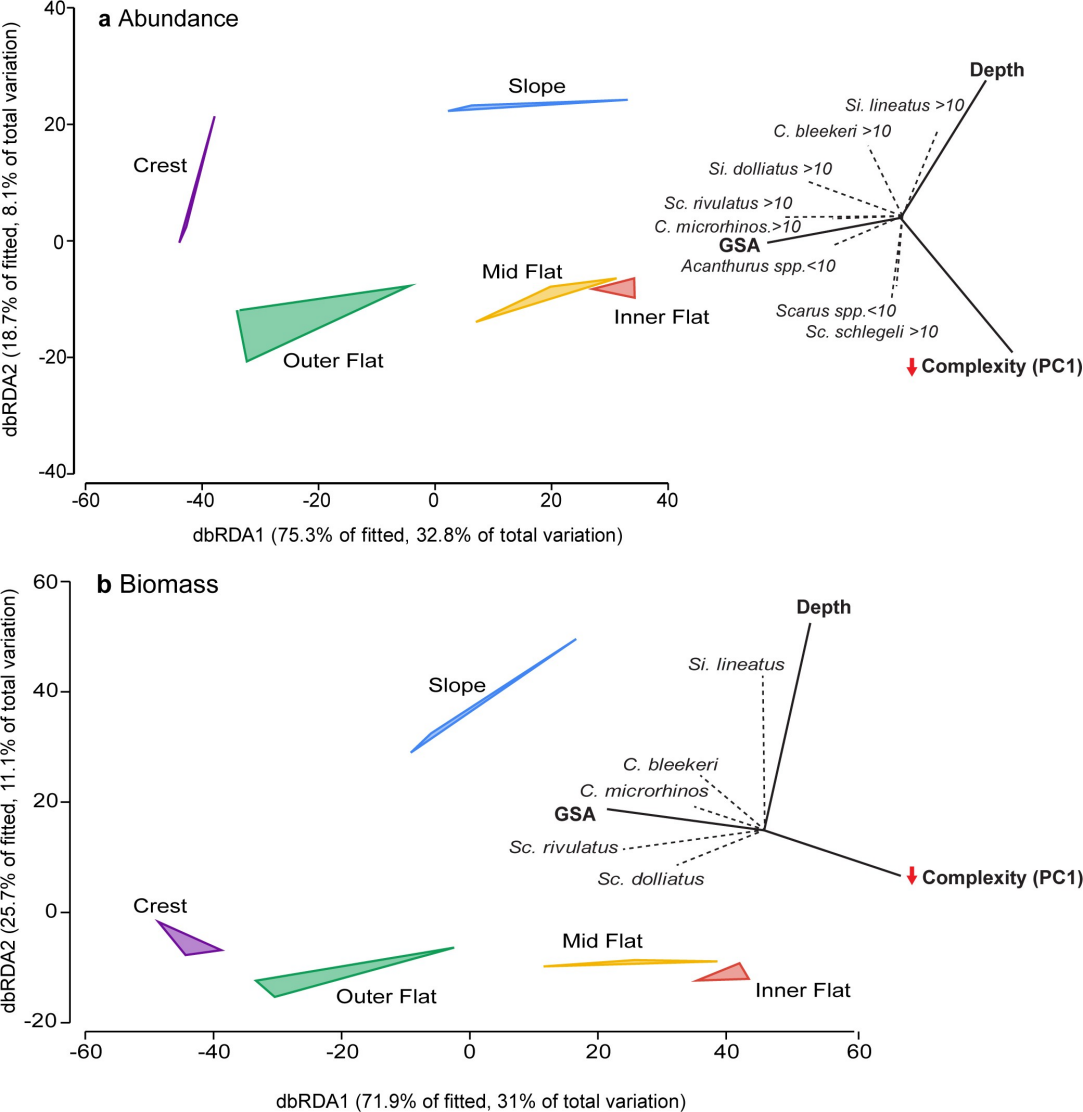
Distance-based redundancy analysis (dbRDA) plots showing the relationship between complexity metrics and nutritional resource availability with the assemblage of herbivorous fishes.

Marginal tests suggested that grazing surface area was significantly correlated with the multivariate data cloud for both abundance and biomass of the fish assemblage (Pseudo-*F* = 2.75, *p* < 0.01; Pseudo-*F* = 3.73, *p* < 0.001, respectively), and explained 17.4% and 22.3% of the total variance in the fish assemblage, respectively (Fig 7). However, the combined complexity metric was only significantly correlated with the species biomass data (Psuedo-*F* = 3.04, *p* < 0.05), explaining 18.9% of the variance, while for the abundance data, complexity only explained 15.6% (Pseudo-*F* = 2.41, *p* = 0.058) (Fig 7). Finally, average water depth was not significantly correlated with either the abundance or biomass of the fish assemblage (Pseudo-*F* = 0.929, *p* = 0.424; Pseudo-*F* = 1.77, *p* = 0.108, respectively), and explained only 0.6% of the variation in abundance data and 11.9% of variation in the biomass data.

The distance-based redundancy analysis plots in terms of **a** abundance and **b** biomass at Orpheus Island. The plots were based on zero-adjusted Bray-Curtis similarity matrices for fish assemblage data. Vectors were calculated using a multiple correlation model. Only vectors with a correlation coefficient >0.3 are displayed. Coloured polygons are to aid visual interpretation and do not denote significant groupings.

## Discussion

Utilising a combination of 3D techniques and traditional ecological methods, this study explored the relationship between a range of habitat complexity metrics and the distribution of roving herbivorous fishes across a depth gradient. The among-habitat distribution of herbivorous fishes documented herein, epitomizes the general patterns documented the world over in disparate biogeographic realms (e.g. [3,7–9]). Specifically, herbivorous fishes (parrotfishes, surgeonfishes, and rabbitfishes) consistently have higher abundances and biomass in shallow-water, relatively high-energy reef zones (the outer-flat or crest). It is widely assumed that habitat complexity is a significant driver of these distributions. However, using high-resolution 3D reconstructions to quantify a wide range of complexity metrics, we revealed that none were good predictors of herbivorous fish distributions across habitats in this system. Instead, availability of nutritional resources was the best predictor of both the abundance and biomass of herbivorous fishes. This departs from the widely held view that complexity/coral cover and fishes are strongly correlated [42,44,75]. It is, however, more in-line with recent evidence that points to a more limited role for coral cover or complexity (e.g. [24,25,53–55]).

Our study highlights how 3D techniques in conjunction with the ‘off-the-shelf’ methods can permit the capture of a benthic dataset that covered a large spatial extent (~ 630 m^2^), to sub-millimetre resolution, with a high degree of accuracy. All of this was achieved with a limited amount of time underwater (~ 124 minutes) and using consumer grade point-and-shoot cameras. This has demonstrated that expensive, hi-tech stereo-vision cameras are not necessary to produce high-resolution 3D models. Moreover, these 3D benthic models permitted the extraction of hi-resolution information that cannot be achieved in-situ because of underwater time constraints (e.g. covering large spatial scales, estimating a fishes’ field of view or allowing for higher levels of replication). Therefore, demonstrating the inherent logistical and practical advantages of these 3D techniques, this study opens new and exciting research avenues to a wider array of researchers so that they may be able to harness the power of this technology in the future, including teasing apart the nature and importance of complexity on coral reefs.

To date, coral reef ecologists have employed a vast array of metrics, across different scales, to characterize complexity (e.g. [16,29,56]). As the broad range of metrics all measure different facets of complexity, one may expect that they will differ markedly from each other. However, contrary to this expectation, when complexity was partitioned to reflect different components, we found complexity covariates were acutely co-linear. Importantly, this high level of collinearity was recorded across all spatial scales examined (10 cm– 5 m), the most relevant scales for the herbivorous fishes examined herein [38].

Notably, our finding of high collinearity between complexity metrics, supports previous studies that have relied on individual metrics such as a RI or coral cover as general proxies for complexity [75,76,77,78]. However, when considered in more detail this high-level of co-linearity is not surprising. One would expect that areas with higher total complexity scores (RI), would also tend to have higher coral cover, as coral calcification directly adds to reef structure [79]. Indeed, this assumption is frequently made in the literature (e.g. [78,80,81]) and supported by previous reviews [12]. While, the relationship of these complexity metrics makes sense, overall complexity does not relate as intuitively to grazing surface area. This was unanticipated, as previous studies have shown that the area, production and removal of algal turfs is generally higher in more complex areas [10,36,82]. In this study system it therefore appears that structural complexity and grazing surface area are unrelated. Importantly, this allowed us to assess the influence of different facets of complexity on the distribution of herbivorous fishes across habitat zones.

Coral reefs are often partitioned into discrete habitat zones, commonly the reef slope, crest and flat. As expected, we revealed that complexity metrics followed a clear pattern, with the highest levels on the slope and crest, and a precipitous decline in complexity across the flat towards the shoreline. However, we revealed that rather than five discrete habitat zones across the reef, there were just three functional zones when characterised based on their structural components. These functional zones highlight the trade-offs that herbivorous fishes must make between having access to nutritional resources while minimizing potential risks associated with predation [13,83,84].

The density of suitable refuges, and the amount of field-of-view can shape the likelihood of a successful predatory attack and the probability that the prey will escape [85]. For instance, experimental manipulations have indicated that holes/crevices are directly related to increases in local prey populations, as they provide suitable areas of refuge [38]. By contrast, Catano et al. [13], demonstrated that while in the presence of a predator decoy, herbivores opted to forage in lower complexity areas, suggesting a preference for enhanced field-of-view to see potential predators. A trade-off clearly exists between having access to suitable refuges to shelter from predation versus being able to see a predator coming sooner, allowing for greater flight initiation distances [38,80,86]. The exact strategy fishes adopt to avoid predation is likely to relate to their biology, as well as the nature of the predators they are trying to avoid [87]. However, it must be noted that while predation risk may be mediated by the characteristics of a given habitat, field of view and access to refugia are just two facets of predation risk. Predation risk can also include predator abundance [88], species-specific behaviours [47] and diurnal predation rates [89], all of which were not encompassed by this study.

There is a broad body of literature that describes a tight linkage between complexity and reef fish abundance (e.g. [16,90,91]). However, we found no clear linkage between complexity and fish abundance or biomass. Instead, herbivorous fish distributions were only related to the availability of grazing surface area. While this finding appears counterintuitive, based on the literature, several previous studies have also noted strong associations between algal-covered benthic surfaces and roving herbivorous fish assemblages [25,44,92]. For example, a series of studies resulting from a multi-decadal ‘natural experiment’ have documented how different reef fish functional groups change in response to varying benthic composition [25,92,93,94]. Notably, abundances of parrotfishes and detritivorous surgeonfishes increased in a strong positive relationship with the coverage of algal turfs following acute disturbances [25,92]. Conversely, ‘off-reef’ planktivourous fusiliers and corallivorous butterflyfishes were particularly sensitive to coral loss, with population densities of both declining markedly with declining live coral cover [93,94]. Furthermore, a recent cross-shelf study on the GBR revealed similar results to the former studies, with disturbance-mediated decreases in live coral cover leading to increases in biomass of herbivorous parrotfishes and surgeonfishes [95].These studies, in conjunction with other studies [26,44,96], highlight that not all functional groups respond equally to losses in coral cover and that particular groups (especially roving nominal herbivores) have strong affiliations with areas high in nutritional resource availability. These associations appear to hold in the present study.

While the influence of grazing area on herbivorous fish distributions is clear, on Orpheus Island, closer scrutiny of the patterns across the three functional zones suggests that field-of-view may also play a role in the high abundance of herbivorous fishes on the outer-flat. This is because, while grazing surface area did not differ significantly across the slope, crest and outer-flat habitats, field-of-view was significantly higher on the outer-flat compared to the crest and slope. This highlights that if grazing surface area was the only factor structuring herbivorous fish distributions, then herbivorous fish populations should have been similar across the three habitats (slope, crest and outer-flat). The key difference (albeit limited to the factors we examined) may be the difference in field-of-view. This suggests that herbivorous fish distributions may be structured by both grazing surface area, and potentially, their ability to see predators coming when utilising this nutritional resource.

The drivers of herbivorous fish distributions are also likely to extend beyond resource availability, as documented in this study. The patterns are probably the result of a complex suite of factors acting in concert. For example, the nature of algal turf resources can vary dramatically among habitats in terms of their productivity, sediment loading and detritus levels [10,97–99]. All such factors can affect herbivorous fish feeding activity [55,100–102] and are also likely to be closely intertwined with habitat complexity [29,103]. Furthermore, hydrodynamic activity such as wave energy [10,104] varies substantially among habitats and can structure fish assemblages. This broad suite of factors highlights the evolutionary obstacles that fishes may have had to overcome to inhabit and exploit particular habitats [4,39]. It also supports recent notions that basic reef geomorphology and habitat are substantial drivers of [105–107], and potentially influenced by [4,39], the nature and structure of herbivorous fish assemblages.

By using a 3D approach to quantify and compare multiple facets of complexity, this study represents one of the first to investigate the influence of various complexity metrics on the distribution of roving herbivorous fishes. In doing so, we revealed minimal linkages between a wide range of traditional complexity metrics and herbivorous fish abundance or biomass at Orpheus Island. Instead, the distribution of herbivorous fishes was strongly associated with the availability of grazing surface area. This suggests that functions such as the removal of algal turfs may continue to operate even in the wake of complexity loss. In the wake of large-scale disturbances and complexity loss, this study offers some cautious optimism for the maintenance of herbivorous fishes on new lower complexity/coral cover reef configurations.

## Supporting information

S1 File Associated figures with the main text. (PDF)

S2 File Associated tables with the main text. (PDF)

S1 Data Raw data. (XLSX)
