# Supplementary material for: Habitat zonation on coral reefs: Structural complexity, nutritional resources and herbivorous fish distributions
Source: PLoS One. 2020 Jun 4;15(6):e0233498. doi: 10.1371/journal.pone.0233498 (PMC7272040; doi:10.1371/journal.pone.0233498)
Supplement: S1 File — (PDF) [file pone.0233498.s001.pdf]

Electronic Supplementary Material

Habitat zonation on coral reefs: structural complexity, nutritional resources and herbivorous fish distributions

Authors:

Arun Oakley-Cogan*, Sterling B. Tebbett, David R. Bellwood

S1 Text Additional figures supporting main text.

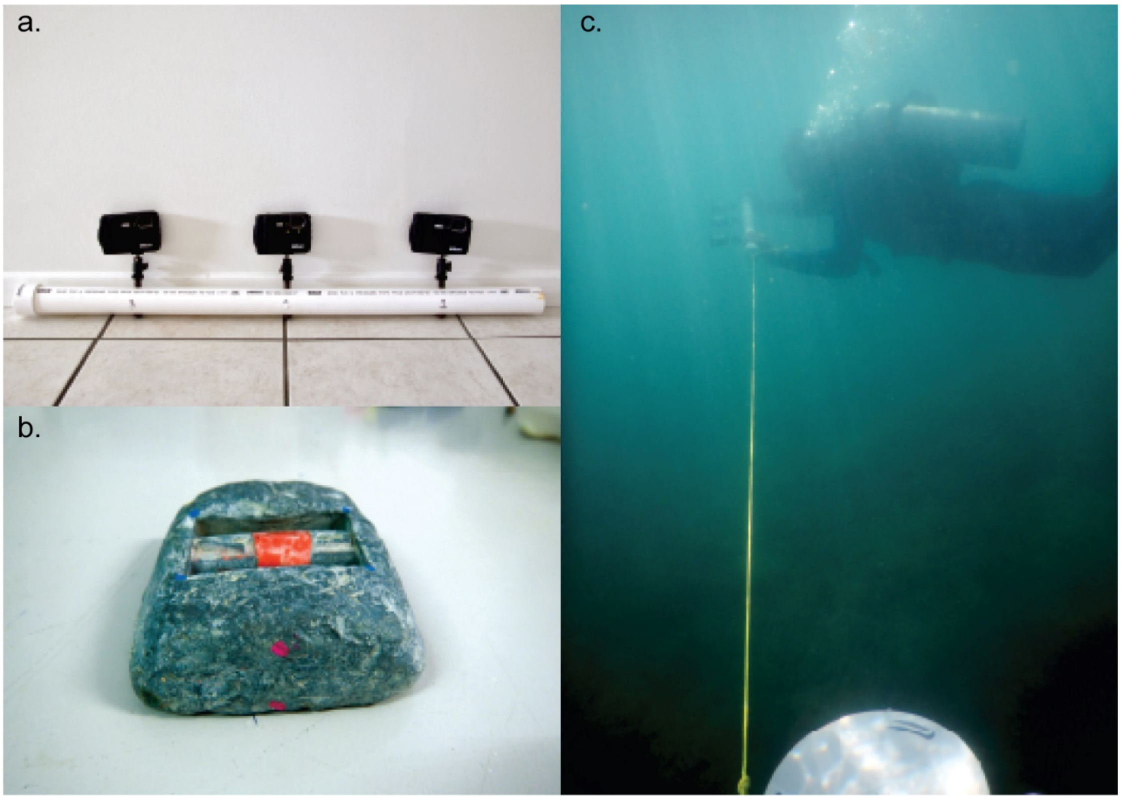

Fig. 1 The custom-built equipment used to capture three-dimensional reconstructions. **a** The custom built camera rig, with three Nikon Coolpix AW300 cameras. **b** Dive weight painted with dots (ground control points) used to scale three-dimensional reconstructions. **c** Surveyor conducting benthic survey with survey-station and attached rig in the foreground.

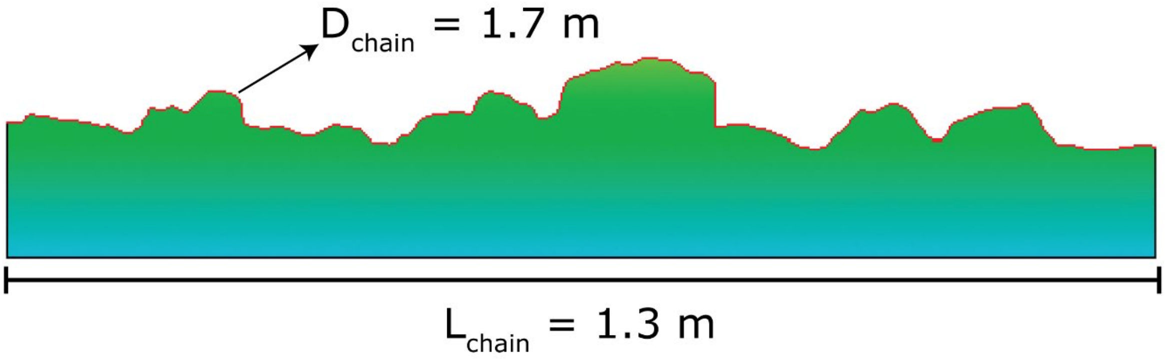

Fig. 2 An example of a virtual 'chain-and-tape' transect subsection

This was used for calculating rugosity index, by relating the linear distance of a chain (L_{chain}) to the distance along a contoured surface (D_{chain}, red line).

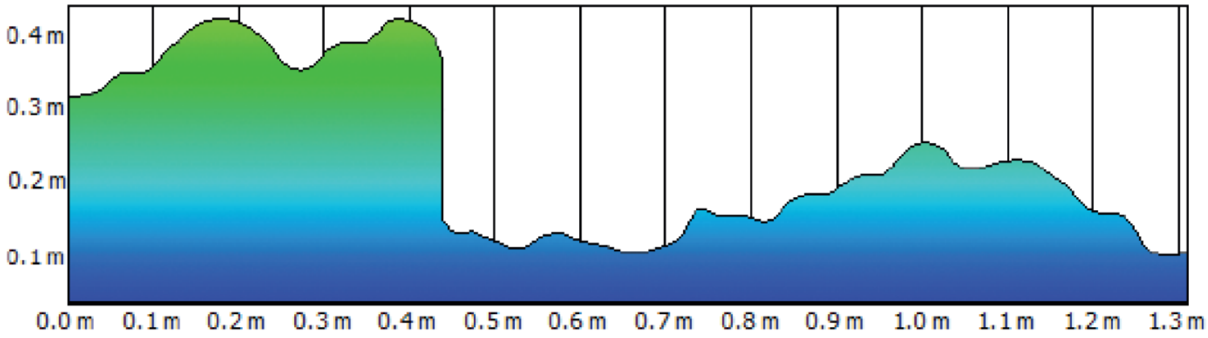

Fig. 3 An example subsection of a reef cross-section used to measure the average rate of change in elevation at 10 cm intervals.

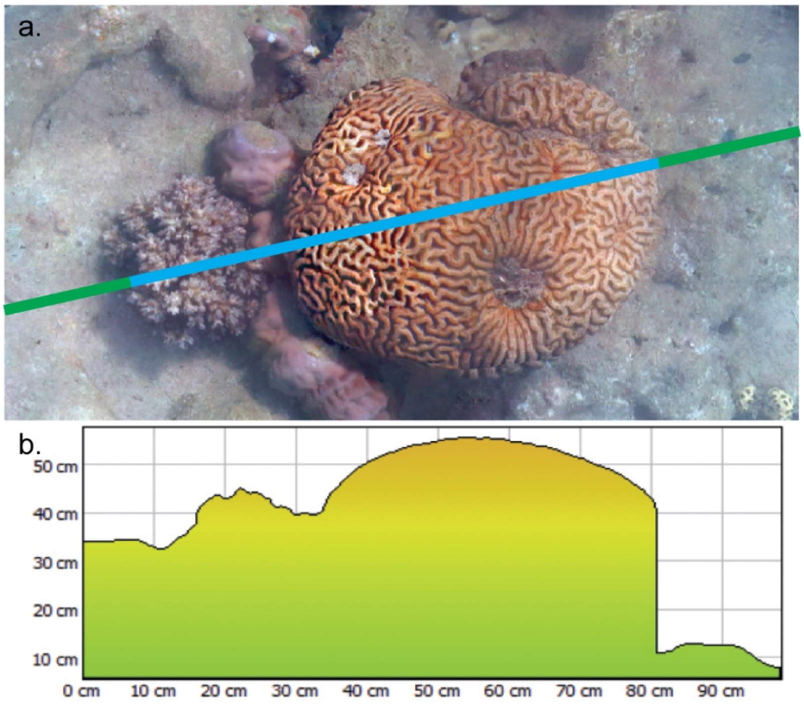

Fig. 4 An example of how coral coverage was categorised along each transect.

a This orthomosaic shows hard coral cover (blue) and grazing surface area (green) and **b** the respective contoured profile used to calculate the proportional coverage.

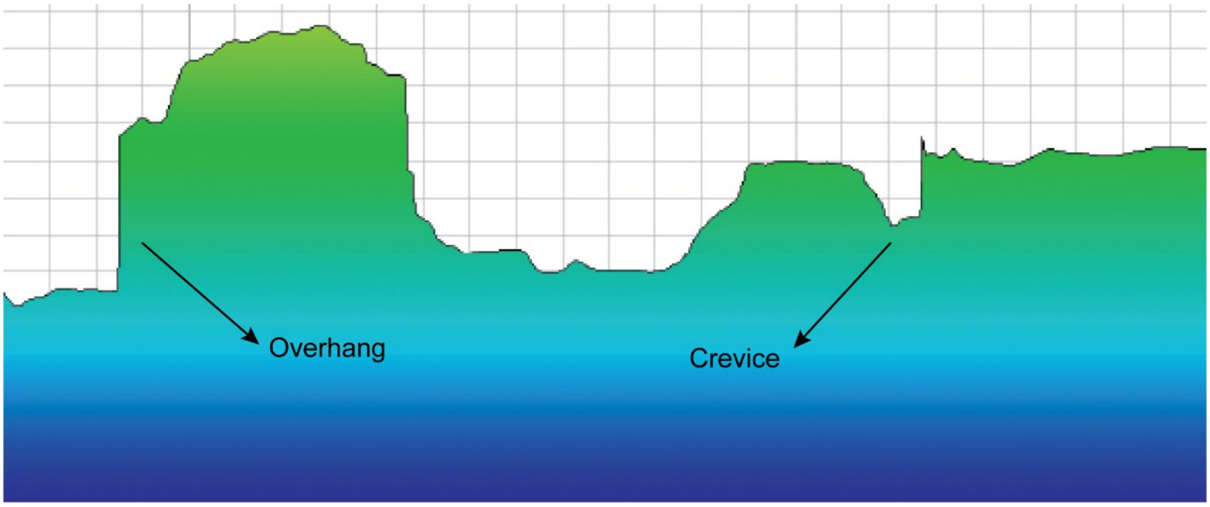

Fig. 5 An example of a reef cross-section, showing examples of overhangs and crevices used to quantify refuge density.

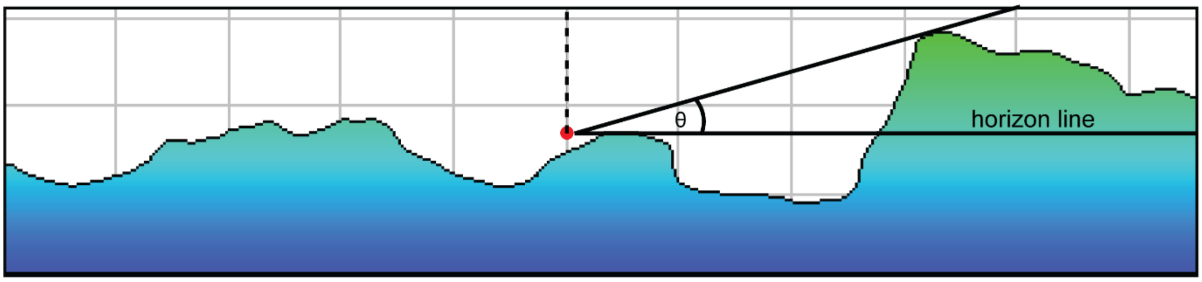

Fig. 6 An example of the methods used to estimate a herbivorous fish's field-of-view.

An observer point (red dot), was placed 2.5 cm above the substratum, the angle was calculated between a horizon line and the hypotenuse connected to the highest topographic point.

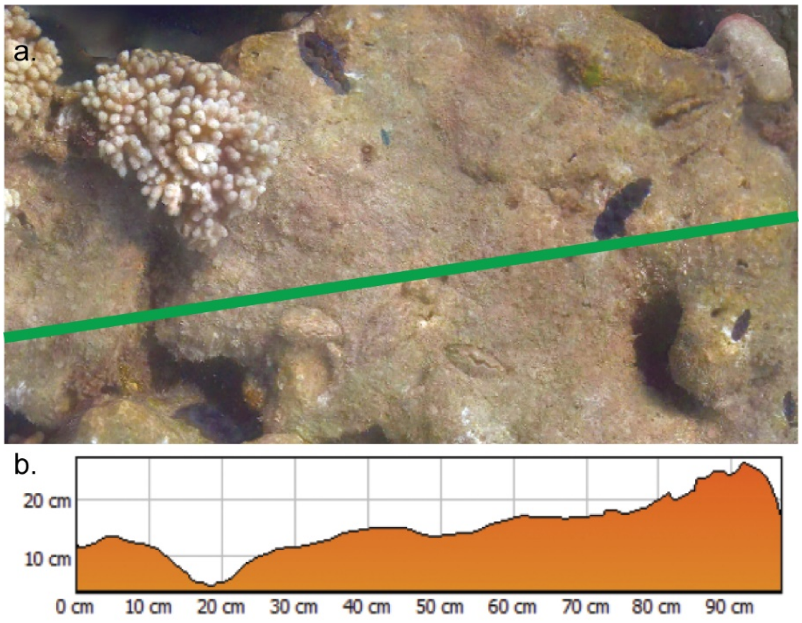

Fig. 7 An example of how the grazing surface area coverage was categorised along each transect.

a This orthomosaic displays grazing surface area (green line) and **b** the respective contoured profile used to calculate the proportional coverage.

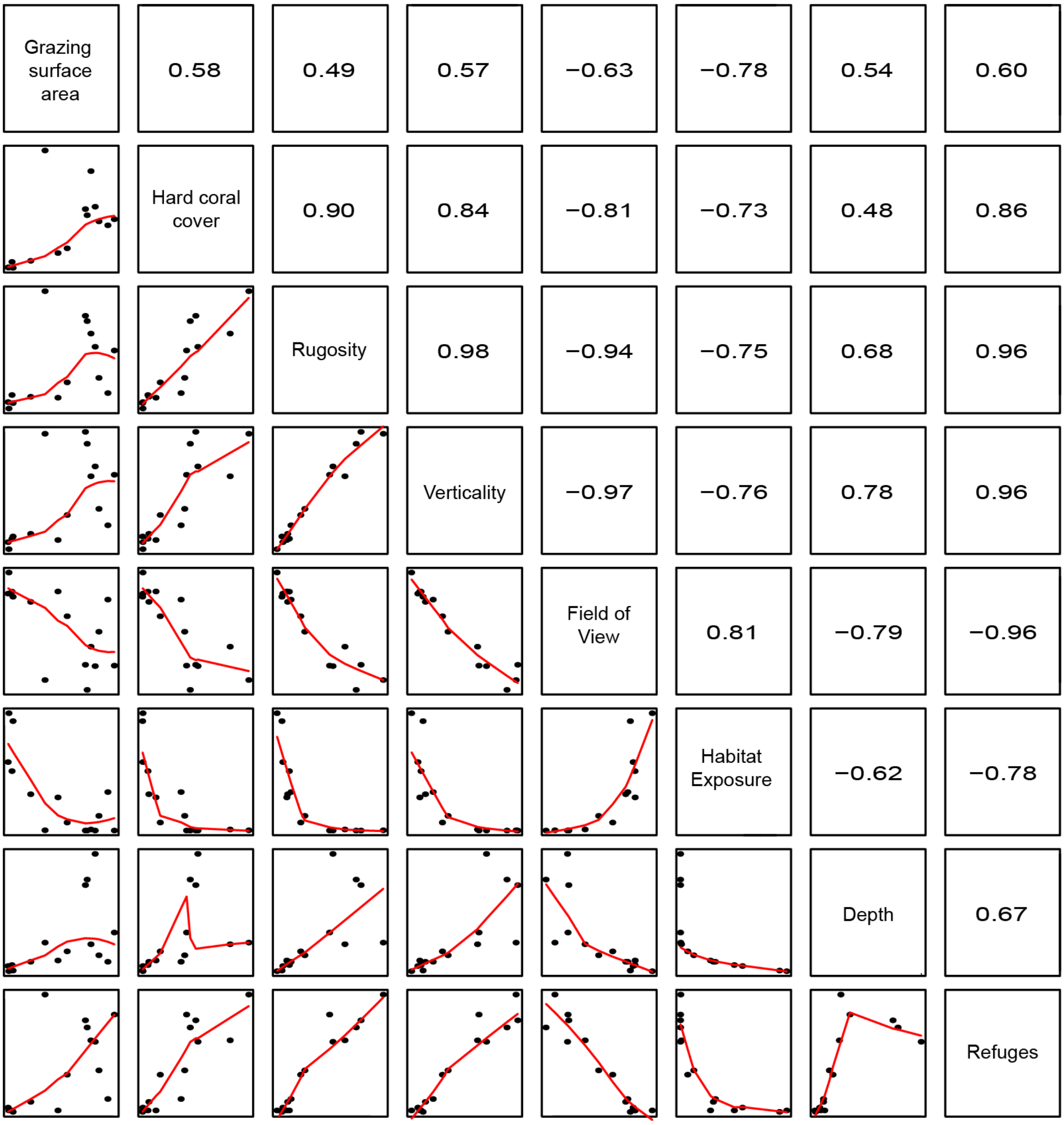

Fig. 8 Scatter plots used to examine collinearity of complexity metrics: rugosity index, verticality, coral cover, refuge density, field-of-view and grazing surface area. Pearson correlation values are given above the diagonal, collinearity of covariates is indicated by Pearson correlation values ± 0.7.

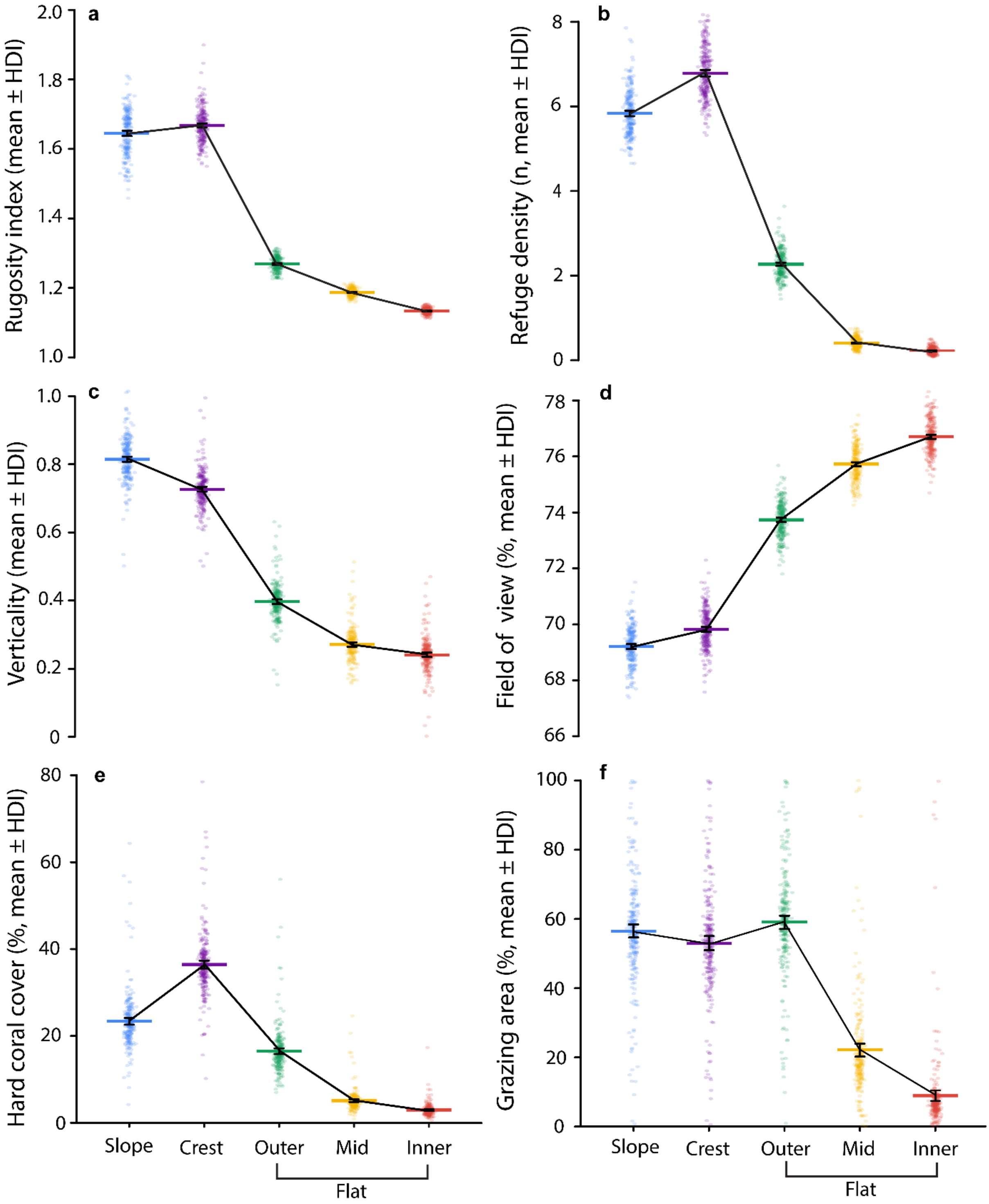

Fig. 9 Complexity metrics along a coral reef depth gradient (reef slope, crest and outer-, mid- and inner-flat) in Pioneer Bay, Orpheus Island. All complexity metrics; **a)** rugosity index, **b)** refuge density, **c)** verticality, **d)** field-of-view, **e)** hard coral cover, and **f)** grazing surface area were quantified using 3D habitat reconstructions. Data points represent a random

sample of 250 draws from the posterior distribution of Bayesian models. Coloured bars represent mean values from posterior distributions and error bars are the lower and upper high posterior density intervals (HPDI).
