# Supplementary material for: Habitat zonation on coral reefs: Structural complexity, nutritional resources and herbivorous fish distributions
Source: PLoS One. 2020 Jun 4;15(6):e0233498. doi: 10.1371/journal.pone.0233498 (PMC7272040; doi:10.1371/journal.pone.0233498)
Supplement: S2 File — (PDF) [file pone.0233498.s002.pdf]

Electronic Supplementary Material

Habitat zonation on coral reefs: structural complexity, nutritional resources and herbivorous fish distributions

Authors:

Arun Oakley-Cogan*, Sterling B. Tebbett, David R. Bellwood

S1 Table Associated tables with the main text

Table 1 Comparison of Bayesian generalised linear mixed effects models used to examine the relationship between the abundance as well as biomass of roving herbivorous fishes and a combined complexity metric (PC1), grazing surface area (GSA) and depth. The most parsimonious model was selected based on the leave-one-out-cross-validation (LOO) criterion. Shown are the expected log pointwise predictive density differences (ELPD Diff) between each model relative to the model with the highest ELPD LOO and the standard error of component-wise differences of ELPD LOO (SE Diff).

Model	Variables	ELPD Diff	SE Diff
Fish abundance	GSA	0.0	0.0
	NULL	-0.8	2.6
	GSA + DEPTH	-1.3	2.4
	DEPTH	-1.8	2.8
	GSA + PC1	-1.9	1.3
	GSA + PC1 + DEPTH	-1.9	2.4
	PC1	-2.4	2.5
	PC1 + DEPTH	-2.8	3.1
Fish biomass	GSA + PC1 + DEPTH	0.0	0.0
	GSA	-0.5	1.7
	GSA + PC1	-0.5	1.0
	GSA + DEPTH	-1.2	1.5
	NULL	-2.2	2.2
	PC1	-2.9	2.2
	DEPTH	-3.6	2.2

PC1 + DEPTH	-3.6	2.1

Table 2 Summary of Bayesian hierarchical models used to compare complexity metrics: rugosity index, verticality and hard coral cover among habitats along a coral reef depth gradient. Bayesian models used 5,000 iterations, 3 chains, a warm-up of 2,500 iterations and a thinning interval of 3, with weakly informative priors. SE = standard error, HPDI = high posterior density interval. If the HDPIs intersected zero no effect was inferred.

**Response variable**	**Model used**	**Predictor variable**	**Estimate**	**SE**	**Lower HPDI**	**Upper HPDI**
Rugosity index	Gaussian (identity link)	Intercept	1.670	0.106	1.510	1.850
		Inner flat	-0.534	0.048	-0.625	-0.437
		Mid flat	-0.480	0.049	-0.571	-0.389
		Outer flat	-0.398	0.051	-0.501	-0.299
		Slope	-0.020	0.077	-0.166	0.137
Verticality	Gaussian (identity link)	Intercept	0.724	0.137	0.485	0.952
		Inner flat	-0.486	0.027	-0.536	-0.430
		Mid flat	-0.456	0.028	-0.511	-0.404
		Outer flat	-0.329	0.029	-0.385	-0.274
		Slope	0.087	0.044	-0.002	0.170
Hard coral cover	Beta-binomial (logit link)	Intercept	-0.585	0.403	-1.490	0.209
		Inner flat	-3.000	0.241	-3.460	-2.510
		Mid flat	-2.430	0.233	-2.890	-1.990
		Outer flat	-1.090	0.200	-1.460	-0.710
		Slope	-0.629	0.186	-0.998	-0.276

Table 3 Summary of pairwise comparisons used to compare complexity metrics: rugosity index, verticality and hard coral cover among habitats along a coral reef depth gradient. SE = standard error, HPDI = high posterior density interval. P = Probability of a difference occurring between contrast levels.

Response variable	Contrast	Estimate	Lower HDPI	Upper HDPI	P
Rugosity index	Crest - Inner flat	0.535	0.437	0.625	1.0
	Crest - Mid flat	0.480	0.380	0.571	1.0
	Crest - Outer flat	0.400	0.299	0.501	1.0
	Crest - Slope	0.020	-0.137	0.166	0.60
	Inner flat - Mid flat	-0.054	-0.080	-0.028	0
	Inner flat - Outer flat	-0.135	-0.174	-0.098	0
	Inner flat - Slope	-0.514	-0.634	-0.395	0
	Mid flat - Outer flat	-0.081	-0.120	-0.041	0
	Mid flat - Slope	-0.459	-0.582	-0.344	0
	Outer flat - Slope	-0.379	-0.507	-0.258	0
Verticality	Crest - Inner flat	0.487	0.430	0.536	1.0
	Crest - Mid flat	0.456	0.404	0.511	1.0
	Crest - Outer flat	0.329	0.274	0.385	1.0
	Crest - Slope	-0.087	-0.170	0.002	0.024
	Inner flat - Mid flat	-0.031	-0.049	-0.014	0.0008
	Inner flat - Outer flat	-0.157	-0.183	-0.134	0
	Inner flat - Slope	-0.574	-0.639	-0.503	0
	Mid flat - Outer flat	-0.127	-0.152	-0.103	0
	Mid flat - Slope	-0.543	-0.610	-0.473	0
	Outer flat – Slope	-0.415	-0.484	-0.342	0
Hard coral cover	Crest - Inner flat	20.015	11.423	30.462	1.0
	Crest - Mid flat	11.263	6.886	17.297	1.0
	Crest - Outer flat	2.962	1.961	4.218	0.674
	Crest - Slope	1.881	1.278	2.640	0.021
	Inner flat - Mid flat	0.566	0.303	0.885	0
	Inner flat - Outer flat	0.149	0.088	0.232	0
	Inner flat - Slope	0.094	0.054	0.145	0
	Mid flat - Outer flat	0.264	0.163	0.409	0
	Mid flat - Slope	0.167	0.097	0.248	0
	Outer flat - Slope	0.627	0.401	0.890	0

Table 4 Summary of Bayesian hierarchical models used to compare complexity metrics: refuge density, field-of-view and grazing surface area among habitats along a coral reef depth gradient. Bayesian models used 5,000 iterations, 3 chains, a warm-up of 2,500 iterations and a thinning interval of 3, with weakly informative priors. SE = standard error, HPDI = high posterior density interval. If the HDPIs intersected zero no effect was inferred.

**Response variable**	**Model used**	**Predictor variable**	**Estimate**	**SE**	**Lower HPDI**	**Upper HPDI**
Refuge	Negative-	Intercept	1.880	0.651	0.879	3.390
density	binomial	Inner flat	-3.440	0.403	-4.240	-2.690
	(log link)	Mid flat	-2.870	0.309	-3.480	-2.320
		Outer flat	-1.110	0.152	-1.400	-0.816
		Slope	-0.144	0.119	-0.358	0.096
Field-of-	Gaussian	Intercept	0.744	0.040	0.669	0.817
view	(identity link)	Inner flat	0.181	0.024	0.136	0.231
		Mid flat	0.163	0.024	0.115	0.210
		Outer flat	0.114	0.024	0.068	0.162
		Slope	-0.022	0.024	-0.069	0.025
Grazing	Beta-binomial	Intercept	0.127	1.080	-2.14	2.21
surface	(logit link)	Inner flat	-2.84	0.248	-3.33	-2.34
area		Mid flat	-1.57	0.228	-2.01	-1.11
		Outer flat	0.270	0.211	-0.125	0.691
		Slope	0.143	0.211	-0.270	0.570

Table 5 Summary of pairwise comparisons used to compare complexity metrics: refuge density, field-of-view and grazing surface area among habitats along a coral reef depth gradient. SE = standard error, HPDI = high posterior density interval. P = Probability of a difference occurring between contrast levels.

**Response variable**	**Contrast**	**Estimate**	**Lower HDPI**	**Upper HDPI**	**P**
Refuge density	Crest - Inner flat	30.551	11.710	65.228	1.0
	Crest - Mid flat	17.556	8.844	30.353	1.0
	Crest - Outer flat	3.033	2.180	3.958	0.765
	Crest - Slope	1.157	0.906	1.427	0
	Inner flat - Mid flat	0.575	0.161	1.257	0.0005
	Inner flat - Outer flat	0.100	0.031	0.186	0
	Inner flat - Slope	0.038	0.012	0.068	0
	Mid flat - Outer flat	0.175	0.078	0.291	0
	Mid flat - Slope	0.066	0.032	0.109	0
	Outer flat - Slope	0.381	0.271	0.502	0
Field-of-view	Crest - Inner flat	-0.181	-0.231	-0.136	0
	Crest - Mid flat	-0.163	-0.210	-0.115	0
	Crest - Outer flat	-0.114	-0.162	-0.068	0
	Crest - Slope	0.022	-0.025	0.069	0.83
	Inner flat - Mid flat	0.017	-0.030	0.064	0.78
	Inner flat - Outer flat	0.067	0.018	0.112	0.99
	Inner flat - Slope	0.203	0.156	0.250	1.0
	Mid flat - Outer flat	0.049	0.003	0.097	0.98
	Mid flat - Slope	0.186	0.136	0.231	1.0
	Outer flat – Slope	0.137	0.090	0.184	1.0
Grazing surface area	Crest - Inner flat	17.094	9.303	26.491	1.0
	Crest - Mid flat	4.775	2.799	7.044	0.99
	Crest - Outer flat	0.763	0.493	1.120	0
	Crest - Slope	0.867	0.533	1.251	0
	Inner flat - Mid flat	0.279	0.156	0.441	0
	Inner flat - Outer flat	0.045	0.024	0.070	0
	Inner flat - Slope	0.051	0.028	0.079	0
	Mid flat - Outer flat	0.159	0.095	0.238	0
	Mid flat - Slope	0.181	0.105	0.267	0
	Outer flat - Slope	1.140	0.703	1.643	0

Table 6 The roving herbivorous fish species observed during visual censuses in Pioneer bay, Orpheus Island.

Family	Taxon name
Acanthuridae	*Acanthurus* spp.
Labridae	*Chlorurus bleekeri*
Labridae	*Chlorurus microrhinos*
Labridae	*Chlorurus spilurus*
Labridae	*Hipposcarus longiceps*
Labridae	*Scarus altipinnis*
Labridae	*Scarus flavipectoralis*
Labridae	*Scarus rivulatus*
Labridae	*Scarus schlegeli*
Labridae	*Scarus* spp.
Siganidae	*Siganus doliatus*
Siganidae	*Siganus lineatus*
Siganidae	*Siganus* spp.

Table 7 Summary of Bayesian hierarchical models used to examine if nominal herbivorous fish abundance and biomass varied among habitats along a coral reef depth gradient. Bayesian models used 5,000 iterations, 3 chains, a warm-up of 2,500 iterations and a thinning interval of 3, with weakly informative priors. SE = standard error, HPDI = high posterior density interval. If the HDPIs intersected zero no effect was inferred.

**Response variable**	**Model used**	**Predictor variable**	**Estimate**	**SE**	**Lower HPDI**	**Upper HPDI**
Abundance	Negative Binomial (log link)	Intercept	-2.090	0.640	-3.300	-0.828
		Inner flat	-1.270	0.556	-2.410	-0.229
		Mid flat	-1.830	0.558	-2.910	-0.714
		Outer flat	1.020	0.538	0.029	2.110
		Slope	-0.810	0.558	-1.930	0.307
Biomass	Gamma (log link)	Intercept	3.13	1.05	1.11	5.13
		Inner flat	-2.69	0.677	-4.02	-1.40
		Mid flat	-5.00	0.683	-6.31	-3.59
		Outer flat	0.017	0.670	-1.26	-1.36
		Slope	-0.847	0.694	-2.10	0.546

Table 8 Summary of pairwise comparisons used to compare nominal herbivorous fish abundance and biomass among habitats along a coral reef depth gradient. SE = standard error, HPDI = high posterior density interval. P = Probability of a difference occurring between the contrast levels.

Response variable	Contrast	Estimate	Lower HDPI	Upper HDPI	P
Abundance	Crest - Inner flat	3.562	0.879	9.074	0.99
	Crest - Mid flat	6.301	1.189	14.785	1.00
	Crest - Outer flat	0.362	0.080	0.884	0.03
	Crest - Slope	2.284	0.502	5.659	0.93
	Inner flat - Mid flat	1.769	0.383	4.427	0.85
	Inner flat - Outer flat	0.100	0.025	0.257	0.00
	Inner flat - Slope	0.642	0.095	1.603	0.21
	Mid flat - Outer flat	0.057	0.013	0.139	0.00
	Mid flat - Slope	0.361	0.061	0.942	0.04
	Outer flat - Slope	6.228	1.355	16.236	0.99
Biomass	Crest - Inner flat	14.70	1.52	44.66	1.00
	Crest - Mid flat	149.00	20.10	455.95	1.00
	Crest - Outer flat	0.99	0.13	2.95	0.49
	Crest - Slope	2.31	0.38	7.21	0.89
	Inner flat - Mid flat	10.10	1.41	31.39	1.00
	Inner flat - Outer flat	0.07	0.01	0.20	0.003
	Inner flat - Slope	0.16	0.02	0.50	0.01
	Mid flat - Outer flat	0.01	0.00	0.02	0.003
	Mid flat - Slope	0.02	0.00	0.05	0
	Outer flat – Slope	2.40	0.26	7.42	0.90

Table 9 Summary of Bayesian hierarchical models used to explore the relationship between nominal herbivorous fish abundance as well as biomass with grazing surface area (GSA). Bayesian models used 5,000 iterations, 3 chains, a warm-up of 2,500 iterations and a thinning interval of 3, with weakly informative priors. SE = standard error, HPDI = high posterior density interval. If the HDPIs intersected zero no effect was inferred.

**Response variable**	**Model used**	**Predictor variable**	**Estimate**	**SE**	**Lower HPDI**	**Upper HPDI**
Herbivore abundance	Gamma (log link)	Intercept	-3.47	0.710	-4.77	-1.97
		GSA	3.01	1.360	4.91	5.81
Herbivore biomass	Gamma (log link)	Intercept	-4.88	0.866	-6.42	-3.07
		GSA	4.77	1.86	1.18	8.21

Table 10 List of water visibility measurements recorded with a secchi disk at the approximate location of each survey site. Measurements were recorded during high tide before the beginning of each survey.

**Date**	**Survey site**	**Visibility (m)**
27/04/2018	1	3.5
28/04/2018	2	3.7
26/04/2018	3	4.4
02/05/2018	1	4.2
30/04/2018	2	3.1
01/05/2018	3	2.8
03/05/2018	1	4.7
02/05/2018	2	4.4
03/05/2018	3	3.9

Table 11 List of average depth measurements for each habitat (n = 3) within each site (n = 3). All measurements were recorded during high tide.

Site	Habitat	Depth (mean, m)
1	Slope	4.12
2	Slope	3.93
3	Slope	5.00
1	Crest	2.31
2	Crest	1.97
3	Crest	1.91
1	Outer Flat	1.31
2	Outer Flat	1.64
3	Outer Flat	1.52
1	Mid Flat	1.20
2	Mid Flat	1.32
3	Mid Flat	1.34
1	Inner Flat	0.98
2	Inner Flat	1.16
3	Inner Flat	1.01

Table 12 List of average habitat exposure times for each habitat (n = 3) within each site (n = 3). Measurements used tide data for the Lucinda (offshore) region (18° 53′ S, 146° 33′ E) and calculated the percentage of time per year that each reef habitat was covered by < 30 cm of water, thereby excluding roving herbivorous fishes from grazing.

Site	Habitat	Exposure (mean, %)
1	Slope	0
2	Slope	0
3	Slope	0
1	Crest	0
2	Crest	0.03
3	Crest	0.14
1	Outer Flat	5.00
2	Outer Flat	1.04
3	Outer Flat	1.91
1	Mid Flat	7.72
2	Mid Flat	4.71
3	Mid Flat	4.30
1	Inner Flat	15.24
2	Inner Flat	8.89
3	Inner Flat	14.21

Table 13 Parameters used in PhotoScan software.

Photo alignment parameters	
Accuracy	High
Key Point limit	40,000
Tie Point limit	4,000
Optimization	
Camera accuracy	10 m
Marker accuracy	0.005 m
Scale bar accuracy	0.001 m
Projection accuracy	0.1 pix
Tie point accuracy	1 pix
Fit f	Enabled
Fit aspect	Disabled
Fit cx, cy	Enabled
Fit skew	Disabled
Fit k1, k2, k3	Enabled
Fit p1, p2	Enabled
Fit k4	Disabled
Dense point cloud	
Quality	Low
Depth filtering	Aggressive
Mesh	
Surface type	Arbitrary
Source data	Dense cloud
Face count	High
Interpolation	Enabled

Table 14 Summary of priors used in Bayesian hierarchical models used to compare complexity metrics and nominal herbivore abundance and biomass among habitats along a coral reef depth gradient. Dpar = distributional parameter.

Response	Prior	Class	Dpar
Rugosity			
	student_t(3,1,10)	Intercept	
	student_t(3,0,10)	Intercept	sigma
	student_t(3,0,10)	sd	
Verticality			
	student_t(3,0,10)	Intercept	
	student_t(3,0,10)	Intercept	sigma
	student_t(3,0,10)	sd	
Field of view			
	student_t(3,1,10)	Intercept	
	student_t(3,0,10)	sd	
	student_t(3,0,10)	sigma	
Refuges			
	student_t(3,1,10)	Intercept	
	student_t(3,0,10)	sd	
	gamma(0.01,0.01)	shape	
Grazing surface area			
	student_t(3,0,10)	Intercept	
	gamma(0.01,0.01)	phi	
	student_t(3,0,10)	shape	
Hard coral cover			
	student_t(3,0,10)	Intercept	
	gamma(0.01,0.01)	phi	
	student_t(3,0,10)	shape	
Herbivore abundance			
	student_t(3,2,10)	Intercept	
	student_t(3,0,10)	sd	
	gamma(0.01,0.01)	shape	
Herbivore biomass			
	student_t(3,2,10)	Intercept	
	student_t(3,0,10)	sd	
	gamma(0.01,0.01)	shape	

Table 15 Summary of priors used in Bayesian hierarchical models used to explore the relationship between nominal herbivorous fish abundance and biomass with grazing surface area (GSA).

Response	Prior	Class
Herbivore abundance		
	student_t(3,-3,10)	Intercept
	student_t(3,0,10)	sd
	gamma(0.01,0.01)	shape
Herbivore biomass		
	student_t(3,-4,10)	Intercept
	student_t(3,0,10)	sd
	gamma(0.01,0.01)	shape
